# Sensitive protein misfolding cyclic amplification of sporadic Creutzfeldt–Jakob disease prions is strongly seed and substrate dependent

**DOI:** 10.1038/s41598-021-83630-1

**Published:** 2021-02-18

**Authors:** Maxime Bélondrade, Simon Nicot, Charly Mayran, Lilian Bruyere-Ostells, Florian Almela, Michele A. Di Bari, Etienne Levavasseur, Joel C. Watts, Chantal Fournier-Wirth, Sylvain Lehmann, Stéphane Haïk, Romolo Nonno, Daisy Bougard

**Affiliations:** 1 Pathogenesis and Control of Chronic Infections, Etablissement Français du Sang, Inserm, Université de Montpellier, Montpellier, France; 2 Department of Food Safety, Nutrition and Veterinary Public Health, Istituto Superiore di Sanita, Rome, Italy; 3 Inserm U 1127, CNRS UMR 7225, UPMC Université Paris 06 UMR S 1127, Institut du Cerveau et de la Moelle épinière, Sorbonne Universités, Paris, France; 4 Tanz Centre for Research in Neurodegenerative Diseases and Department of Biochemistry, University of Toronto, Toronto, Canada; 5 IRMB, INM, INSERM, CHU Montpellier, (LBPC-PPC), Univ Montpellier, Montpellier, France

**Keywords:** Prions, Protein folding, Diseases of the nervous system, Laboratory techniques and procedures, Diseases

## Abstract

Unlike variant Creutzfeldt–Jakob disease prions, sporadic Creutzfeldt–Jakob disease prions have been shown to be difficult to amplify in vitro by protein misfolding cyclic amplification (PMCA). We assessed PMCA of pathological prion protein (PrP^TSE^) from 14 human sCJD brain samples in 3 substrates: 2 from transgenic mice expressing human prion protein (PrP) with either methionine (M) or valine (V) at position 129, and 1 from bank voles. Brain extracts representing the 5 major clinicopathological sCJD subtypes (MM1/MV1, MM2, MV2, VV1, and VV2) all triggered seeded PrP^TSE^ amplification during serial PMCA with strong seed- and substrate-dependence. Remarkably, bank vole PrP substrate allowed the propagation of all sCJD subtypes with preservation of the initial molecular PrP^TSE^ type. In contrast, PMCA in human PrP substrates was accompanied by a PrP^TSE^ molecular shift during heterologous (M/V129) PMCA reactions, with increased permissiveness of V129 PrP substrate to in vitro sCJD prion amplification compared to M129 PrP substrate. Combining PMCA amplification sensitivities with PrP^TSE^ electrophoretic profiles obtained in the different substrates confirmed the classification of 4 distinct major sCJD prion strains (M1, M2, V1, and V2). Finally, the level of sensitivity required to detect VV2 sCJD prions in cerebrospinal fluid was achieved.

## Introduction

Prion diseases or transmissible spongiform encephalopathies (TSEs) are fatal neurodegenerative disorders affecting humans and other mammals. They are characterized by long asymptomatic incubation periods followed by a short symptomatic stage in which a progressive and severe alteration of brain functions is associated with spongiform changes, neuronal loss, and gliosis at the neuropathological level^1,2^. Human prion diseases have an annual incidence of about 1–2 cases per million people worldwide^3^ and, unlike other proteinopathies, occur in sporadic (85% of cases; sporadic Creutzfeldt–Jakob disease-sCJD), genetic (10–15% of cases; genetic CJD, Gerstmann Sträussler–Scheinker Syndrome, Fatal Familial Insomnia) and acquired (less than 5%; Kuru, iatrogenic CJD, variant CJD) forms^4^. According to the protein-only hypothesis^5^, TSEs are associated with the autocatalytic conversion of the cellular prion protein (PrP^C^), a glycoprotein with a high proportion of alpha-helices, into an abnormal beta-sheet enriched isoform (PrP^TSE^)^6^. This conversion process may involve a seeding/nucleation activity that relies on the ability of preformed prion aggregates to trigger the aggregation of their normal counterparts. These structural changes lead to the accumulation of PrP^TSE^ in the form of amyloid fibers forming inconstant but characteristic plaques in brain tissues of affected individuals^7^. Among different TSEs, various pathophysiological profiles in terms of incubation time, duration of illness, lesion profile, or clinical presentation can be observed. Such phenotypic diversity is believed to be driven by different isoforms of aggregated PrP^TSE^, or strains, which contain specific pathological information that is transmitted during the prion conversion process^8^. In human TSEs, the phenotypical diversity strongly relies on a methionine/valine polymorphism at codon 129 of human *PRNP* gene (M129V) and on the distinctive biochemical properties of PrP^TSE^ such as glycoform ratio (i.e. variable di-, mono-, and unglycosylated isoforms) or size of the proteinase K-resistant prion protein core, termed PrP^res^. Proteinase K (PK) digestion allows the classification into two types of PrP^res^, types 1 and 2, distinctive by the electrophoretic mobility of the unglycosylated component at either 21 or 19 kDa^9^. Considering sCJD patients, all *PRNP* codon 129 genotypes are represented. By combining the genotype with the PrP^res^ isoform, sCJD patients can be classified into six major subtypes: MM1, MM2, MV1, MV2, VV1 and VV2 in which each of them are generally associated with a specific clinico-pathological phenotype^10^. MM1 and MV1 cases are considered as a single subtype (MM/MV1) as they are phenotypically similar while MM2 cases include two different clinico-pathological phenotypes: the cortical (MM2C) and the thalamic types (MM2T)^11^. However, variability in the biochemical methods used to distinguish PrP^TSE^ types as well as the presence of mixtures of PrP^res^ types in more than 30% of sCJD patients (MM1 + 2, MV1 + 2 or VV1 + 2) complicates the classification of human sporadic prions^12–15^. In the last decade, new approaches based on the seeding/nucleation property of prions have emerged for TSEs diagnosis. Two main techniques have been developed to artificially reproduce this seeding activity in vitro: Protein Misfolding Cyclic Amplification (PMCA)^16^ and Real Time-Quaking Induced Conversion assay (RT-QuIC)^17^. These approaches allow the aggregation process to occur in a few days in contrast to the years-timeframe observed in individuals incubating the disease. RT-QuiC has been shown to be highly specific and sensitive for the diagnosis of sCJD or gCJD in the CSF or in nasal brushing samples but is less effective in detecting vCJD prions^18,19^. On the other hand, PMCA has been used for the specific diagnosis of vCJD in urine, blood or CSF^20–26^, while it is sparsely mentioned in the literature as a powerful method to amplify sCJD prions^27–34^. Exploiting our experience in vCJD prion amplification using PMCA, we evaluated here different PrP^C^ sources as substrate for the amplification of sCJD isolates of the 6 different subtypes^10^. We evaluated the suitability of human PrP^C^ protein, 129 M or 129 V, expressed in transgenic mice. In order to complement the data obtained with human PrP substrates, we also included bank vole PrP as a PMCA substrate. Indeed, bank voles and transgenic mice expressing bank vole PrP have been shown to be highly susceptible to prion strains from different species, including different sporadic, genetic and acquired human prion diseases^35–40^. Furthermore, the bank vole PrP has been shown to be a suitable substrate for the efficient in vitro amplification of human prions^32,33,41^. Here we demonstrate efficient and substrate-dependent PMCA of sCJD prions that showed in vitro seeding behaviors consistent with previously established classifications. In addition, the sensitivity of our PMCA allowed the detection of PrP^TSE^ in the CSF of patients with sCJD of different subtypes.

## Results

### Efficient amplification of sCJD is substrate dependent

**Figure 1:**
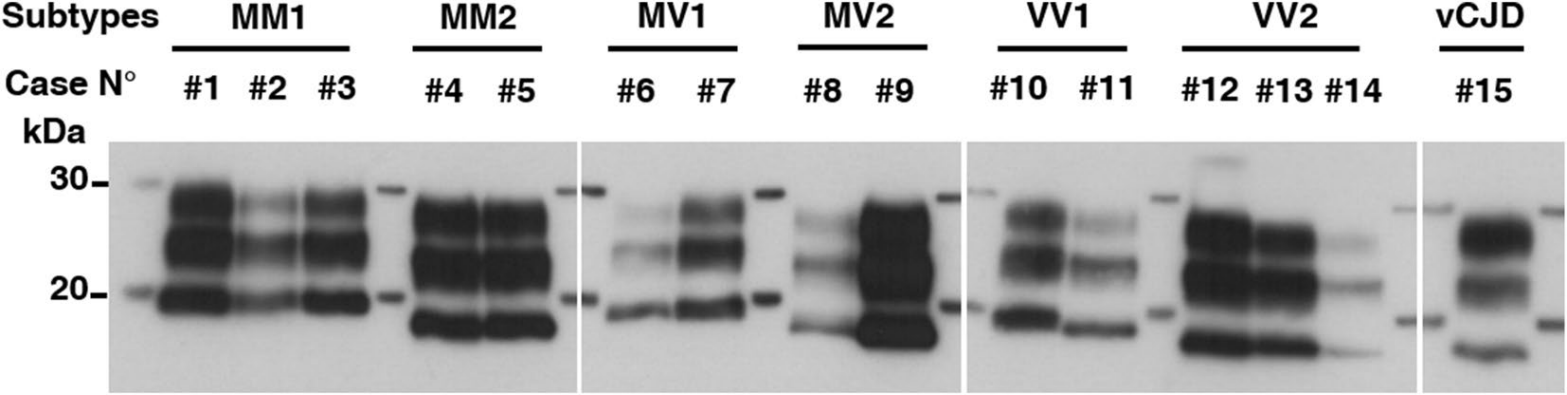
Western blot analysis of non-amplified brain samples. A panel of 14 brain samples (#1–#14) from patients with sCJD was obtained from the French CJD National Surveillance Network. #15 corresponds to vCJD reference brain sample provided by the NIBSC. The PrP^TSE^ signal was assessed by means of western blot analysis using 3F4 antibody after proteinase K digestion. For each sample, the equivalent of 20 µL of 0.1% (w/v) brain homogenate was loaded onto the gel.

**Figure 2:**
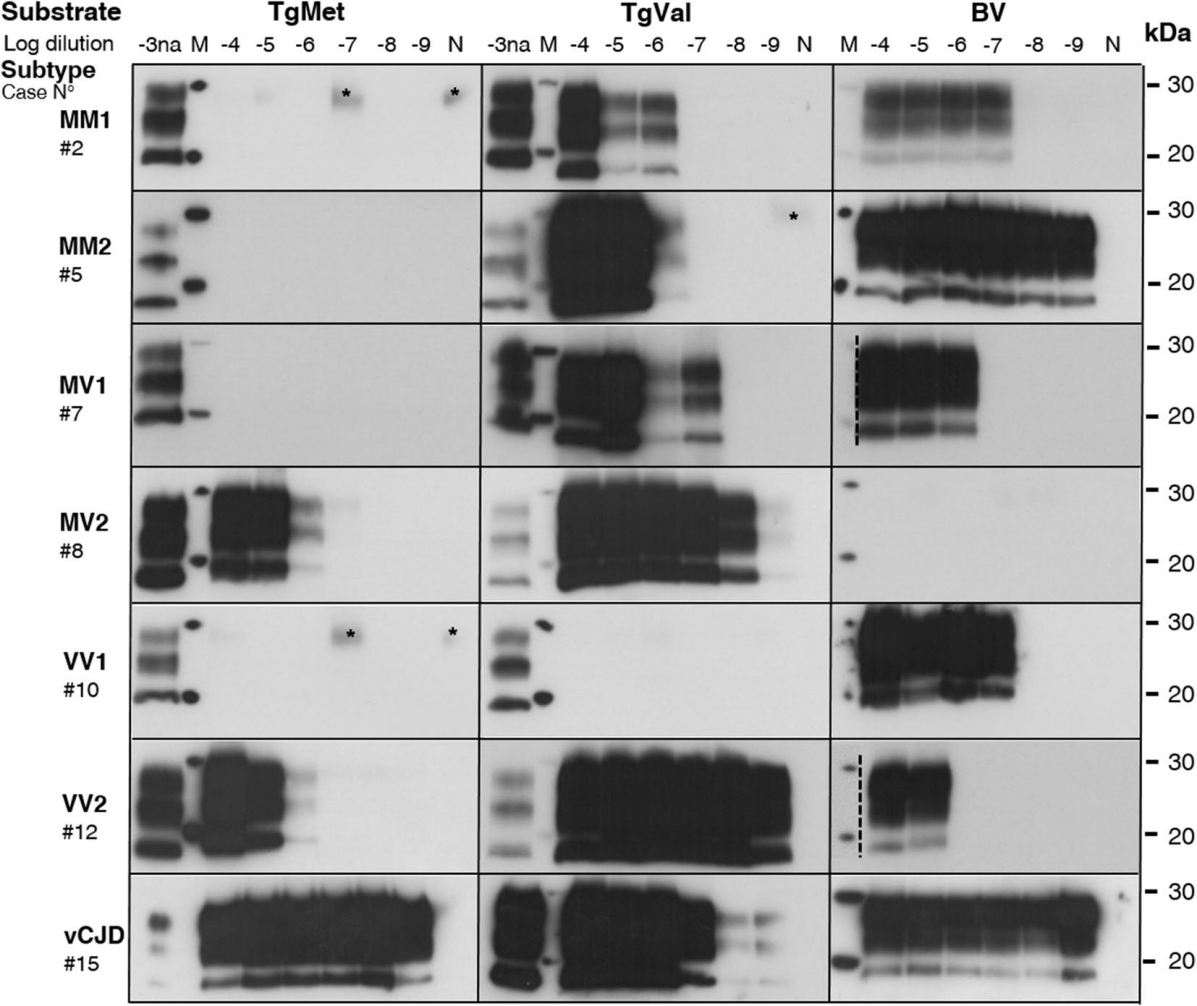
PMCA representative level of amplification for each different sCJD subtype in TgMet, TgVal and BV substrates. Serial dilutions from 10^−4^ to 10^−9^ of the different sCJD subtypes in addition to vCJD were amplified using TgMet, TgVal or BV substrates. After 4 rounds of PMCA, the PrP^TSE^ signal was assessed by western blot analysis after proteinase K digestion using 3F4 antibody for TgMet and TgVal amplicons and 6D11 Ab for BV amplicons. For each sample, 20 µL of the product was loaded onto the gel. −3na refers to non-amplified material (no PMCA) obtained from a 10^−3^ dilution (w/v) of the initial infectious brain sample. N refers to substrate only amplified in the same conditions. The asterisk indicates a faint signal from incomplete PrP^C^ digestion. M indicates the typical molecular mass of PrP^res^ in the range of 20–30 kDa. Hatched lines correspond to cropped images.

Infected brain homogenates (IBH) from 14 patients with confirmed sCJD of MM1 (#1–3), MM2 (#4,5), MV1 (#6,7), MV2 (#8,9), VV1 (#10,11), or VV2 (#12–14) subtype were used as PrP^TSE^ seeds for the PMCA reaction and compared against the vCJD WHO reference case from the NIBSC (#15) (Table 1). In Fig. 1, western blot analysis of the non-amplified materials (10^−3^ dilution w/v) from each IBH is represented as an illustration of the different molecular profiles type 1/type 2 PrP^res^ of each subtype as well as their initial PrP^res^ level. Serial PMCA amplification of each IBH were performed using three different substrates referred to as TgMet, TgVal and BV hereafter in presence of heparin as a cofactor. Dilutions from 10^−4^ to 10^−9^ were submitted to serial PMCA and PrP^res^ generated after 4 rounds was analyzed by western-blot. The number of 4 rounds was fixed based on vCJD previous data for which dilution limit of brain homogenate was obtained after 3 PMCA rounds therefore a plateau in the amplification could be expected after 4 rounds. PMCA overall results are summarized in Table 1 and representative PrP^res^ signals are illustrated in Fig. 2. Seeding efficiencies varied in a subtype and substrate dependent manner. No PrP^res^ signal was observed after PMCA in all the unseeded substrates (N; n = 45). For each discordant results, experiments were repeated three times.

**Table 1:** Summary of PMCA results after 4 rounds according to seed/substrate combination (dilutions tested from 10^−4^ to 10^−9^).

IBH subtype	TgMet substrate		TgVal substrate		BV substrate	
	Detection limit	PrP^res^ type	Detection limit	PrP^res^ type	Detection limit	PrP^res^ type
**MM1**						
#1	–		–		10^−6^	1
#2	–		10^−6^	2	10^−7^	1
#3	–		10^−6^	2	10^−7^	1
**MM2**						
#4	–		10^−5^	2	**10**^**−9**^	2
#5	–		10^−6^	2	**10**^**−9**^	2
**MV1**						
#6	–		10^−6^	2	10^−5^	1
#7	–		10^−7^	2	10^−6^	1
**MV2**						
#8	10^−6^	1	**10**^**−9**^	2	–	
#9	10^−7^	1	**10**^**−9**^	2	10^−8^	2
**VV1**						
#10	–		–		10^−7^	1
#11	–		10^−4^	2	–	
**VV2**						
#12	10^−6^	1	**10**^**−9**^	2	10^−5^	2
#13	10^−7^	1	**10**^**−9**^	2	10^−4^	2
#14	10^−6^	1	**10**^**−9**^	2	–	
**vCJD**						
#15	**10**^**−9**^	2	**10**^**−9**^	2	**10**^**−9**^	2

Bold indicates that the last 10-9 dilution tested was positive.

After 4 rounds of PMCA, vCJD amplification was achieved in all 3 substrates with detection of the 10^−9^ dilution (last dilution tested). Although sCJD MM1 prions were unable to convert TgMet, 2 out of 3 cases could seed the PMCA reaction in TgVal with moderate efficiency, down to a 10^−6^ dilution. Amplification using BV NBH was efficient and consistent down to the 10^−6^/10^−7^ dilutions. Optimal amplification of sCJD MM2 was achieved in BV (10^−9^ dilution) while no or moderate amplification was obtained in TgMet and TgVal respectively. Prions from sCJD MV1 and MV2 patients were preferentially amplified in TgVal, allowing the detection of the 10^−6^/10^−7^ and 10^−9^ dilutions, respectively. As with sCJD MM1 and MM2 prions, TgMet did not amplify MV1 prions. Moderate amplification of MV1 was obtained in BV, allowing the detection of the 10^−5^/10^−6^ dilutions. Regarding sCJD MV2 prions, PMCA in the TgMet substrate allowed the detection of the 10^−6^/10^−7^ dilutions and discordant results were obtained in BV. No amplification for one case (#8) and amplification allowing detection down to the 10^−8^ dilution with the second case (#9) were obtained with this substrate. These results were confirmed in two additional series of PMCA experiments. The sCJD VV1 prions were the most difficult to amplify in our conditions: no amplification in TgMet, very limited amplification (10^−4^) in TgVal for only one case (#11) and unexpectedly higher amplification in BV (10^−7^ dilution for case #10) but with again discordant results between VV1 cases. In Fig. 1, the two VV1 cases showed slight differences in the PrP^res^ size which could support the marked differences in PMCA amplification. Signals shown in Fig. 2 in TgVal and BV are from the same sCJD VV1 case (#10).

Unlike the other sCJD subtypes, VV2 prions were easier to amplify and like vCJD, could be amplified in all 3 substrates, except case #14 in BV but PrP^res^ level in this brain tissue was lower compared to the others (Fig. 1). The best substrate for the sCJD VV2 amplification was TgVal allowing the detection of the 10^−9^ dilution and the least effective was BV allowing detection only up to 10^−4^/10^−5^ dilutions.

The efficient amplification of sCJD MM1 prions in BV prompted us to challenge overexpressing bank vole PrP using TgBV substrate. Unfortunately, the seeding potential of sCJD MM1 was not increased in TgBV compared to the wild type BV, (detection of the 10^−6^ dilution) (see Supplementary Fig. S1).

### Amplified PrP^res^ could shift according to the PMCA substrate

**Figure 3:**
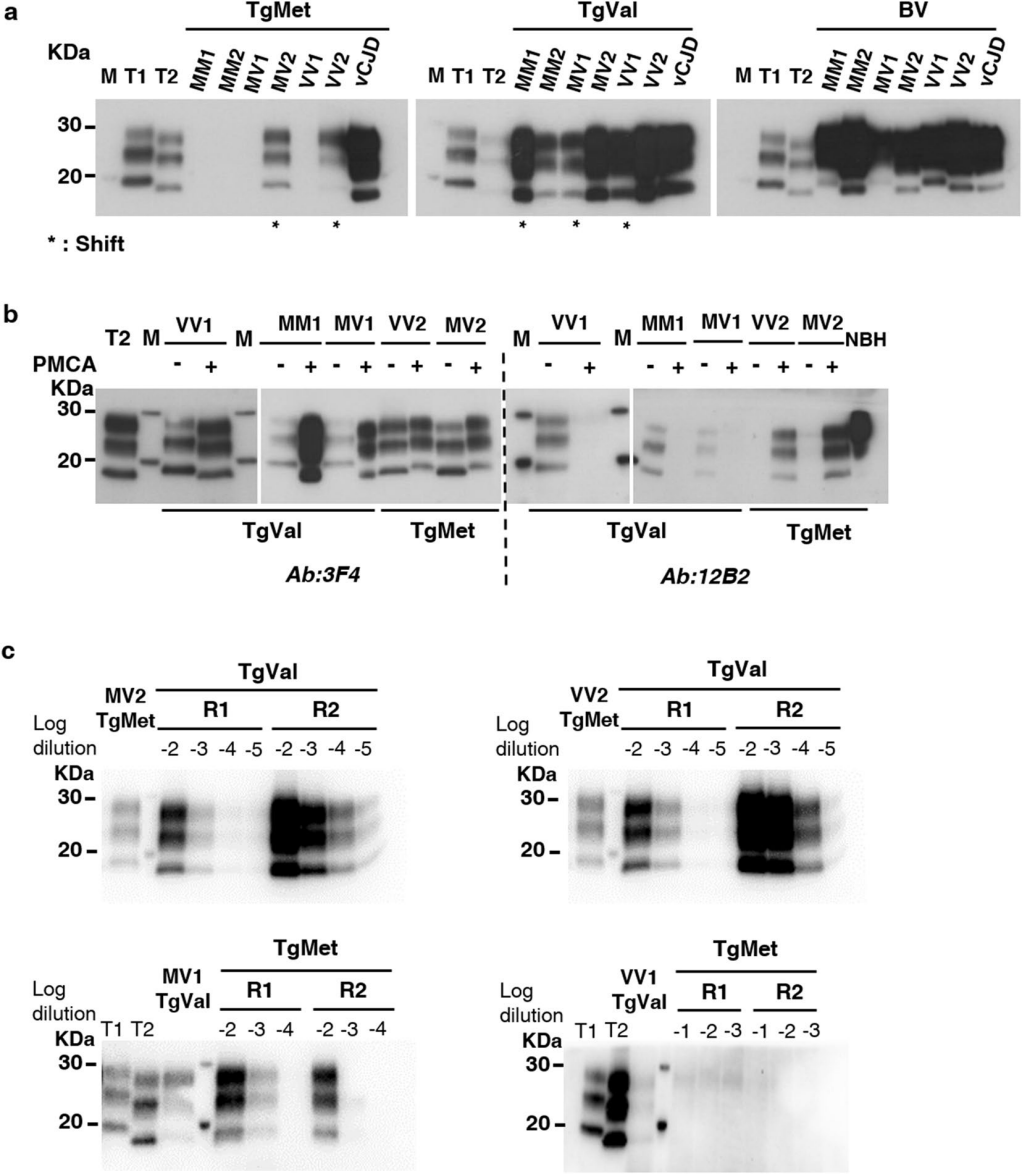
PrP^res^ shift after PMCA of sCJD according to the substrate. sCJD subtypes as well as vCJD prions were amplified during 4 rounds of PMCA with TgMet, TgVal and BV substrates. The PrP^TSE^ signal was assessed by western blot analysis after proteinase K digestion. T1 corresponds to non-amplified type 1 PrP^res^ (21 kDa) from a 10^−3^ dilution of either sCJD MM1 or MV1 IBH. T2 corresponds to non-amplified type 2 PrP^res^ (19 kDa) from a 10^−3^ dilution of either sCJD VV2 or MV2 IBH. M indicates the typical molecular mass of PrP^res^ in the range of 20–30 kDa; (**a**) PrP^res^ molecular profile after PMCA of sCJD in the different substrates. * indicates generated amplicons with a molecular type1/type2 PrP^res^ shift compared to the initial seed; antibody 9A2; (**b**) PrP^res^ electrophoretic profiles of shifting PMCA amplicons (+) and their corresponding initial seeds (−) were compared using 3F4 Ab (recognizing both type1 and type2 PrP^res^) and 12B2 Ab (specific for type1 PrP^res^). NBH refers to normal brain homogenate from TgMet mice without any proteinase K digestion. For (**a**) and (**b**), loaded materials are from different seeded dilutions and have been adjusted in order to better visualize unglycosylated bands: in TgMet: 10^−4^ for MM1, MM2, MV1 and VV1; 10^−5^ for MV2 and VV2, 10^−8^ for vCJD; in TgVal: 10^−4^ for MM1, MM2, and VV1, 10^−5^ for MV1 and vCJD, 10^−7^ for MV2 and VV2; in BV: 10^−4^ for VV2, 10^−5^ for MV1, 10^−6^ for MM1 and VV1, 10^−7^ for MM2, MV2 and vCJD. (**c**) Back-seeding of MV2/VV2 amplified in TgMet using TgVal substrate and MV1/VV1 amplified in TgVal using TgMet substrate. MV2 and VV2 PMCA amplicons generated in TgMet substrate (lane 1) were serially diluted from 10^−2^ to 10^−5^ and amplified back in TgVal substrate for 2 rounds (R1 and R2). Similarly, MV1 and VV1 PMCA amplicons generated in TgVal substrate (lane 3) were serially diluted from 10^−2^ to 10^−4^ for MV1 or from 10^−1^ to 10^−3^ for VV1 and amplified back in TgMet substrate for 2 rounds (R1 and R2); antibody: 3F4.

Independently of amplification efficiency of IBH/substrate couples, we exploited the 9A2 antibody, which recognizes both human and bank vole PrP, to examine by western blot (WB) the specific electrophoretic profiles of the different PMCA amplicons according to the substrate used. When type 2 subtypes IBH (MV2 and VV2) seeded TgMet substrate or when type 1 subtypes IBH (MM1, MV1 and VV1) seeded TgVal substrate_,_ a molecular type1/type2 shift of the unglycosylated isoform of PrP^res^ was observed (Fig. 3). Amplicons generated from BV substrate conserved their electrophoretic typing profiles compared to the initial IBH used to seed the reaction (Fig. 3a).

To assess whether the observed shift was due to a modification of the proteinase K cleavage site, we analyzed TgMet or TgVal shifting PMCA amplicons using 3F4 Ab (epitope 109–112 of human PrP) and 12B2 Ab (epitope 89–93 of human PrP, specific for type 1 PrP^res^) (Fig. 3b). After 4 rounds of PMCA amplification using TgMet substrate, type 2 subtypes shifted to a type 1 profile, which was confirmed by the presence of a WB signal using 12B2 Ab on generated amplicons. In contrast, type 1 subtypes shifted to a type 2 profile after PMCA in TgVal, as confirmed by the absence of WB signal using 12B2 Ab.

To explore the dynamic property of shifting amplicons, we performed a back-seeding PMCA using the shifted PMCA amplicons as seeds. We first used MV2/VV2-TgMet amplicons as seeds back into TgVal substrate (Fig. 3c). Serial dilutions of the amplicons from 10^−2^ to 10^−5^ were submitted to 2 PMCA rounds. Type 2 profiles were recovered in TgVal substrate with efficient seeding potential (10^−5^ dilution detected after 2 rounds). Thus, while amplification of the VV2 subtype in TgMet substrate led to a type 1 amplicon, its seeding activity back in TgVal was completely different to the pure Type 1 VV1 subtype which failed to seed or poorly seeded the TgVal substrate (#10 and #11 in Table 1). These findings thus highlight a reversible molecular PrP^res^ signature of MV2/VV2 sCJD prions upon PMCA conversion in human PrP M129 substrate without apparent alteration of their seeding properties. We next complemented this data by performing a reverse experiment, in which MV1/VV1-TgVal amplicons (shifted to type 2) were used to seed the TgMet substrate (Fig. 3c). The MV1-TgVal amplicons harbored a low seeding potential in the TgMet substrate (detection of only the 10^−2^ dilution after 2 rounds), accompanied with a back shift into type-1 PrP^res^. This effect was not observed with direct seeding of PMCA reactions with the sCJD MV1 subtypes in TgMet (Table 1), highlighting that PMCA in TgVal allowed the MV1 prions to acquire a moderate seeding potential back into the TgMet substrate; however this seeding effect was clearly lower from that of the pure MV2 sCJD subtype in the TgMet substrate (#8 and #9 in Table 1). By contrast, the VV1-TgVal could not seed back the TgMet substrate (Fig. 3c). Taken together, these findings are in agreement with the notion that the observed PrP^res^ shifts during the heterologous PMCA in the human PrP substrates are not associated with major changes in the seeding properties of sCJD prions, although in the case of MV1 prions some slight modifications could be observed during back seeding experiments.

### Electrophoretic signatures of CJD PMCA amplicons concord with the existence of four strains of sCJD

Seeding behaviors of IBH/substrate couples were consistent with previously described observations from in vivo models (Table 1). In addition to presenting a similar amplification potential (10^−6^–10^−7^ dilutions detected after 4 rounds of PMCA), VV2 and MV2 subtypes harbored a type 1 electrophoretic profile in TgMet substrate (shift to type1), while maintaining a type 2 profile when seeded in TgVal substrate with a maximal amplification efficiency (i.e. detection of the 10^−9^ dilution). These two subtypes could be considered as a same strain of prion, i.e. the V2 strain^42^.

Regarding MM1 (#2 and #3) and MV1 subtypes, similar behaviors were shown and are reminiscent of the M1 prion strain. Indeed, with a positive amplification down to the 10^−6^ dilution, the PMCA amplicons obtained in TgVal substrate adopted a type 2 PrP^res^ profile (Fig. 3a; Table 1) and no amplification was observed in TgMet substrate. Both MM1 and MV1 subtypes could be amplified in BV substrate with, however, a better seeding activity of MM1 (detection of the 10^−6^/10^−7^ dilutions) compared to MV1 (detection of the 10^−5^/10^−6^ dilutions).

When analyzing the amplification of different sCJD subtypes in BV substrate, a specific feature appeared for MM2 subtype with a maximum level of amplification with this subtype (detection of the 10^−9^ dilution).

VV1 subtype also displayed a unique behavior as no, poor, or variable amplification was obtained with all of the substrates tested in our PMCA settings.

Amplification results obtained by PMCA in this study fit well with the previously proposed classification of M1 (MM1 and MV1), V2 (VV2 and MV2), M2 (MM2) and V1 strain (VV1), on the basis of the seeding efficiency with the different substrates and shifting properties of PrP^res^ from the PMCA amplicons.

### PMCA amplification allowed the detection of sCJD prions in CSF samples

**Figure 4:**
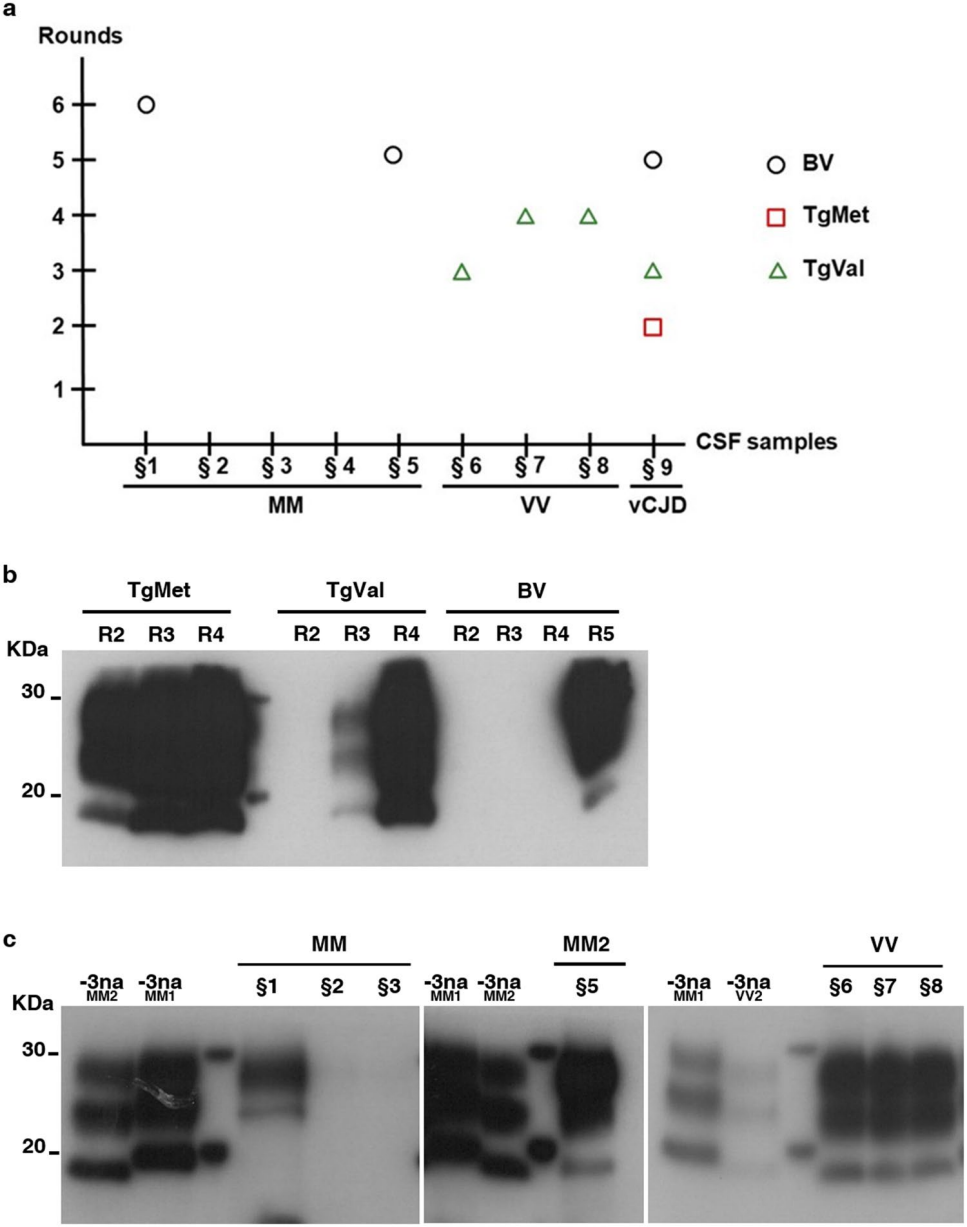
Detection of PrP^TSE^ in the CSF of CJD patients. CSF samples from 8 sCJD patients (§1–§8) and 1 definite vCJD patients were serially amplified by PMCA. The PrP^TSE^ signal was assessed by means of western blot analysis after proteinase K digestion using 9A2 antibody. For each sample, 20 µL of the product were loaded onto the gel. R refers to the number of rounds. M indicates the typical molecular mass of PrP^res^ in the range of 20–30 kDa. (**a**): Representation of the results according to CSF sample genotypes and number of rounds; (**b**): vCJD amplification in TgMet, TgVal and BV substrates; (**c**): sCJD amplification: MM subtypes in BV substrate; VV subtype in TgVal substrate. − 3na MM2/MM1/VV2 refer to non-amplified material (no PMCA) obtained from the 10^−3^ dilution (w/v) of the initial infectious brain samples from MM2, MM1 and VV2 subtypes, respectively.

CSF samples (n = 9) collected from patients with probable or definite CJD were submitted to four to six serial PMCA rounds using the most adapted substrate. All patients were homozygous at codon 129: 5 MM genotypes including 2 definite MM1 cases and one MM2 confirmed case, three VV genotypes including one definite VV2, and one definite vCJD case. According to our abovementioned results obtained using IBH, BV was used to amplify MM genotype, TgVal for VV genotype and TgMet, TgVal and BV for vCJD samples. Results are summarized in Fig. 4. Generated PMCA amplicons were analyzed by WB using 9A2 Ab. As previously shown^20^, vCJD amplification from CSF samples using TgMet is efficient with a positive signal observed from the second round. While TgVal and BV also supported vCJD amplification from CSF samples, 3 and 5 PMCA rounds, respectively, were required to detect a PrP^res^ signal (Fig. 4b). Among the 5 sCJD-MM cases analyzed, only two (§1 and §5) gave a positive result after prolonged PMCA rounds (i.e. 5 rounds for the sCJD-MM2 case (§5) and 6 rounds for the MM probable sCJD case §1). WB analysis of PMCA amplicons showed a type 2 PrP^res^ preservation for sample §5 from MM2 subtype (Fig. 4c). The three CSF samples from patients with probable or definite VV sCJD (§6 to §8) could seed the PMCA reaction in TgVal substrate and gave a positive PrP^res^ signal after three or four rounds. Considering the very low PMCA efficiency to amplify the VV1 subtype with TgVal substrate, we could assume that the two probable VV sCJD cases were likely of VV2 subtype.

## Discussion

A major issue in TSE therapeutics research is the antemortem characterization of the prion agent/strain involved that would allow CJD patient stratification in clinical trials. This is of special interest since the efficacy of anti-prion compounds may vary according to strain^43^, including CJD strains^44^. Recently, new in vitro approaches, based on the seeding properties of pathological prion aggregates were developed for the diagnosis of human TSEs. For example, RT-QuIC is a very sensitive and specific assay for the diagnosis of sCJD and is about to be implemented worldwide in neurology-specialized health care facilities^45,46^. In the present study, we provide a comprehensive analysis of the PMCA seeding activity across the spectrum of sCJD subtypes, using three different brain substrates derived from humanized PrP transgenic mice (methionine or valine homozygous at codon 129) and bank voles. Contrary to the vCJD prions, obtaining an efficient PMCA of sCJD prions was not as straightforward. That is why we became interested in cofactors, specifically heparin that had been used as a powerful enhancer of prion amplification by PMCA^47^. Based on its common usage, we have added heparin in all of the PMCA reactions described in this study.

Overall, we found major differences in the seeding properties of sCJD subtypes. Depending on the seed/substrate PMCA pairing, a shift in the electrophoretic mobility of the generated PrP^res^ amplicons was observed in some cases. This heterogeneity highlights some important in vitro differences in the capacity of sCJD subtypes to seed PMCA reactions in a substrate-dependent manner.

According to the sensitivity of the PMCA and the PrP^res^ electrophoretic profiles obtained using 3 different substrates, we were able to classify the different sCJD subtypes into 4 distinct groups, i.e. MM1/MV1, MM2, VV1, and MV2/VV2. This is in agreement with the previously proposed existence of 4 distinct sCJD prion strains based on bioassay transmission studies in human transgenic mice, bank voles and non-human primates^35,48–50^, as inferred from the analysis of molecular characteristics of PrP^TSE^ in vitro^12,51^ or by combining both approaches^51^ and referred to as M1, M2, V1 and V2 respectively.

Our data obtained with human transgenic mice also support the notion that the PrP^C^ polymorphism at codon 129 from the substrate can directly influence the PrP^res^ electrophoretic profile obtained after PMCA, sometimes leading to a shift (type-1 or type-2) from the initial PrP^res^ signature. In TgMet brain substrate, both MV2 and VV2 sCJD acquired a type-1 PrP^res^, whereas in TgVal substrate, MM1, MV1 and VV1 sCJD sources, when amplified, shifted towards a type-2 PrP^res^. These observations are in partial accordance with the results of previous in vivo studies. Bishop et al. have shown that transmission of MV2 and VV2 sCJD subtypes to HuMM mice (expressing physiological levels of human PrP homozygous for methionine at codon 129) was accompanied by a type-1 PrP^res^ shift in mouse brains^48^. A similar in vivo observation was also reported after transmission to the same TgMet mice used in our study^49^ and confirmed by Cassard et al*.* after the propagation of MV2 and VV2 sCJD prions from the brain of 10 different patients with confirmed sCJD in a very similar TgMet line^14^. However, in Bishop et al. study completed by the recent results from Cassard et al., type-1 sCJD subtypes transmitted to HuVV mice maintained their initial type-1 PrP^res^ signature, which in this respect contrasts with our current in vitro observations. Using cell-based PMCA, Takeuchi et al*.* have shown that atypical CJD-MM1 cases, harboring plaque lesions, when amplified with substrate containing Valine129 PrP, gave type 2 amplicons^29^. Interestingly, at least for the V2 sCJD strain (MV2 and VV2), these phenotypic modifications were not accompanied by major changes in the initial seeding characteristics, as illustrated by the strong ability to replicate back in the TgVal substrate in the form of type-2 PrP^res^. The V2 strain amplified in TgMet substrate, while acquiring a type 1 profile, differed clearly from the V1 sCJD strain, which amplified very poorly in the TgVal substrate. This is reminiscent of the traceback phenomenon observed in vivo by passaging sCJD V2 prions to 129 M then 129 V human PrP-expressing mice^52^.

In contrast to the shift observed with PMCA in humanized transgenic mice substrates, our study revealed that amplification of different sCJD types in the bank vole (Met_109_) substrate, in addition to sustaining human prion amplification in almost all cases tested, allowed a faithful conservation of the initial PrP^res^ type present in the brain of the sCJD patients. This contrasts with a change from type-1 to type-2 PrP^res^ reported by Redaelli et al. after PMCA amplification of MM1 sCJD in bank vole substrate^33^. Our results are however in good agreement with previous bioassay transmission studies in either wild-type bank voles^35^ or transgenic mice overexpressing bank vole PrP (both Met_109_)^36^, from which MM1 and MV1 sCJD propagate as type-1 PrP^res^ and MM2, VV2 or vCJD propagate as type-2 PrP^res^. However, regarding glycosylation ratio, another hallmark of PrP^TSE^ characterization, we noticed a systematic augmentation of the diglycosylated isoforms in all amplicons generated by PMCA, whatever the initial seed used (from IBH or CSF) or the substrate used. Using human prion strains, this modification in the glycosylated ratio from mono- to diglycosylated isoforms was previously mentioned from in vivo^35,36,38,53^, in vitro^33^ or ex vivo^54^ results.

The level of amplification obtained in the TgMet substrate in our study was somewhat disappointing, especially in the homologous PMCA context for MM1 and MM2 prions. Indeed the compatibility of the genotype at codon 129 between the seed and substrate has been proposed to be one of the most important factors for an efficient amplification^27^. Also, MM1 sCJD prions were transmitted very efficiently in TgMet mice^14,55^, which contrasts with our in vitro observations. Our failure to amplify MM1 and MM2 sCJD prions with TgMet substrate could be due to prion strain characteristics that were not fully compatible with the PMCA settings (sonication power and/or duration, time of incubation steps), and were thereby not efficiently sustaining the seeding of PMCA reactions, presumably in the early steps (initiation/elongation kinetics). It could be due also to an intrinsic inability of the Met129 PrP (TgMet) to support in vitro MM1 prion amplification, however, the same substrate allowed the amplification of vCJD very efficiently and repeatedly^56^, and to a lesser extent also the V2 sCJD strain. It is worth noting that, considering the PMCA outcome using substrates containing human PrP (TgMet and TgVal), we obtained the best amplification efficiency for V2 strain (VV2 and MV2) and vCJD which show the highest stability with regards to PK digestion^57^.

Given the encouraging PMCA amplification potential obtained for certain sCJD types in specific substrates and in order to demonstrate the feasibility of using PMCA on easily-accessible peripheral tissues or body fluids, we tested a small panel of CSF samples collected at clinical stage from patients with probable or definite sCJD. These included 5 MM cases that were tested in the bank vole substrate, 3 VV cases tested in TgVal substrate, as well as 1 vCJD case tested in all 3 substrates, which served as a positive control and was previously detected blindly using TgMet substrate^20^. For vCJD prion detection in CSF, the TgMet substrate appeared to be the most efficient, although positive results were obtained using TgVal and bank vole substrates after prolonged PMCA rounds.

Most importantly, we could detect a PMCA seeding activity in some of the sCJD CSF samples analyzed. Detection of sCJD PrP^TSE^ from CSF samples by PMCA had been previously described by Rubenstein et al*.*^31^, however their specific PMCA setting associated to Surround Optical Fibre ImmunoAssay (SOFIA) as readout precluded sCJD subtypes differentiation. Regarding the seeding activity from the five CSF from MM patients, efficiency was variable as only two out of five cases were detected positive after 5/6 PMCA rounds. Our PMCA failed to identify CSF samples from the two definite MM1 sCJD patients. Considering RT-QuIC studies, the analytical sensitivity required to detect a seeding activity in the CSF of MM1 patients might be around the 10^−8^ dilution of the brain samples. This level is not achieved in this study even by using bank vole PrP as substrate. Nevertheless, the CSF sample from a definite MM2 patient seeded the PMCA reaction in BV substrate and gave a positive type 2 signal after 5 PMCA rounds. Like PrP^TSE^ of brain origin, PrP^TSE^ from CSF samples displayed similar seeding behavior in BV substrate with regards to type 1/2 preservation. sCJD in CSF samples from VV patients was consistently detected using TgVal substrate after 3–4 PMCA rounds. These results indicate that all three VV sCJD patients were probably of VV2 subtypes, the second most common sCJD subtype after MM1.

In conclusion, this study demonstrates the potential of PMCA for the sensitive amplification of sCJD prions across the spectrum of human sCJD subtypes, which showed marked seed/substrate amplification heterogeneities. Noteworthy, PMCA could accurately discriminate between 4 sCJD prion strains thus recapitulating the current in vivo classification, establishing PMCA as an efficient in vitro model of sCJD prions propagation. For some prion strains—notably the V2 strain—the level of sensitivity achieved suggests that PMCA might be amenable to sCJD prion detection in peripherally accessible samples.

## Methods

### Brain tissues

Brain tissue obtained at autopsy from sporadic or variant CJD cases came from either the UK National Institute for Biologicals Standards and Control (NIBSC) CJD Resource Centre in the form of 10% (w/v) homogenate in 0.25 M sucrose (WHO reference NHBYO/0003 for vCJD) or from the French CJD National Surveillance Network as a block of frontal cortex tissue. The diagnosis was confirmed neuropathologically. All six currently defined subtypes of sCJD (MM1-MM2-MV1-MV2-VV1-VV2) were represented (2–3 different patients for each subtype—MM2 are of MM2 cortical type) (see Supplementary Table S1). Infected brain homogenates (IBH) at 20% (weight/volume) in 5% glucose were prepared using a high-speed homogenizer (MiniBeadbeater).

### CSF samples

CSF samples were provided by the National CJD Research and Surveillance Unit, United Kingdom and by the university hospital (CHU) of Montpellier, France. The CSF had been collected in polypropylene tubes under standardized conditions. The samples were transferred to one laboratory within 4 h of being collected and centrifuged at a rate of 1000*g* for 10 min at 4 °C. It was then aliquoted into 0.5-mL polypropylene tubes and stored at − 80 °C for further analysis. CJD cases were classified as sporadic or variant CJD (definite or probable) using internationally recognized criteria^58^ (see Supplementary Table S2).

### PMCA

Normal PrP used as substrate was obtained from two humanized transgenic mouse lines: TgMet overexpressing sixfold the level of human PrP with a methionine at codon 129 (Tg650 line)^55^ and TgVal overexpressing four–eight-fold the level of human PrP with a valine at codon 129 (Tg152 line)^59^; wild type bank vole (BV) carrying methionine at codon 109^35^; and bank vole transgenic mice (TgBV) overexpressing 4.9 fold the level of bank vole PrP with a methionine at codon 109 (Tg22019±)^60^. After collection, brains were rinsed in cold PBS and immediately frozen on dry ice before long-term storage at − 80 °C. Normal brain homogenates (NBH) were prepared at 10% (weight: volume) in conversion buffer (phosphate-buffered saline containing 150 mM sodium chloride, 1% Triton, protease inhibitor cocktail (Roche), EDTA 1 mM) and clarified at 2000×*g* for 20 s before freezing at − 80 °C in single-experiment aliquots.

For amplification by PMCA, 10 µL of the different infected brain homogenates (IBH) serially tenfold diluted (10^−4^–10^−9^ w/v) or 20 µL of CSF were mixed with 90 µL of PMCA substrate supplemented with heparin at 10 µg/mL (Sigma) in PCR-tubes containing three Teflon beads (diameter 2.388 mm; Marteau & Lemarié). Unseeded substrates were also included in each PMCA experiments as Negative controls. Each PMCA cycle is composed of an incubation step (14 min 40 s at 37 °C) and a sonication step (20 s at 240 W). Successive rounds of 160 cycles were performed by diluting the amplified material in fresh heparin-supplemented PMCA substrate (1:10 for serial dilutions and 1:5 for CSF). For IBH dilutions, 4 amplification rounds were applied. CSF samples were amplified in up to 6 rounds of PMCA. To avoid any cross-contamination, experiments were carried out under strict quality control PCR conditions.

### Proteinase K (PK) digestion and SDS–PAGE/immunoblotting

After amplification, protease-resistant prion protein was detected by western blot as described previously^61^. After proteinase K digestion (200 µg/mL) for 60 min at 45 °C and denaturation at 100 °C in SDS–PAGE denaturing buffer, samples were run on 12% polyacrylamide gel electrophoresis, before being electro-transferred onto PVDF membrane. Western blot (using the SNAP ID system, Millipore) was performed using 3F4 (mAb 3F4, epitope 109–112 of human PrP—Ozyme, France), 12B2 (mAb 12B2, epitope amino acid residues 89–93 of human PrP—Wageningen Bioveterinary, Netherlands), 9A2 (mAb 9A2, epitope amino acid residues 99–101—Wageningen Bioveterinary, Netherlands) or 6D11 (mAb 6D11, epitope 93–109 of human PrP sequence—Ozyme, France), and anti-mouse IgG peroxidase-linked secondary antibody (GE Healthcare, UK) linked to a chemiluminescent reaction (ECL blotting detection reagent, GE Healthcare, France), and imaged using films except for Fig. 3c using the imaging system Fusion FX7 (Vilber, France). The detection limit was determined visually after a maximum time exposure of 30 min and as result the dilution scored positive when the three characteristic PrP^res^ bands were observed.

### Ethics statement

The human CSF and brain samples used in this study were provided by the Laboratory of Clinical Proteomics (Montpellier, France) and the French National Center of Reference for Prions (Paris, France). A written informed consent for autopsy and research use was provided by patients’ relatives, according to French legislation (L.1232-1 to L.1232-3, Code de la Santé Publique). Collection, preservation and preparation of human samples for research purpose have been declared to the French Ministry of Research (number DC-2008-417 and DC-2009-957) according to French regulation (L.1243-3 and L. 1243-49, Code de la Santé Publique). A few CSF samples were also provided by the NCJDRSU (UK) with informed consent obtained from the next of kin for research use (05/MRE00/67). All experiments on human samples were carried out in accordance with French regulation (L.1243-3 and L. 1243-49, Code de la Santé Publique) and all protocols were approved by the EFS research committee and supervised by the Scientific Direction of ANSM (Project No. P69).

All the animal experiments made to collect the brains from mice or bank voles at euthanasia (using carbon dioxide) were carried out in strict accordance with EU directive 2010/63. Mouse experiments were carried out in strict accordance with the recommendations from the Guide for the Care and Use of Laboratory Animals, as provided by the French Ministry of Agriculture and of the European Union (project authorization number 02298.03 provided by the French Ministry of Research after ethical evaluation). Bank voles were obtained from the breeding colony of Istituto Superiore di Sanità (ISS). The experimental protocol was approved and supervised by the Service for Biotechnology and Animal Welfare of the Istituto Superiore di Sanità and authorised by the Italian Ministry of Health (Decree No. 1119/2015-PR). All procedures were carried out in accordance with European Council directives 86/609 and 2010/63, as well as in compliance with the Italian Legislative Decree 26/2014.

## Supplementary Information

Supplementary Information.
