# Supplementary material for: Sensitive protein misfolding cyclic amplification of sporadic Creutzfeldt–Jakob disease prions is strongly seed and substrate dependent
Source: Sci Rep. 2021 Feb 18;11:4058. doi: 10.1038/s41598-021-83630-1 (PMC7893054; doi:10.1038/s41598-021-83630-1)

SUPPLEMENTARY INFORMATION

Sensitive Protein Misfolding Cyclic Amplification of Sporadic Creutzfeldt-Jakob disease Prions is Strongly Seed and Substrate Dependent

Maxime Bélondrade[1], Simon Nicot[1], Charly Mayran[1], Lilian Bruyère-Ostells[1], Florian Almela[1], Michele A. Di Bari[2], Etienne Levavasseur[3], Joel C. Watts[4], Chantal Fournier-Wirth[1], Sylvain Lehmann[5], Stéphane Haïk[3], Romolo Nonno[2], Daisy Bougard[1*]

[1]Pathogenesis and control of chronic infections, Université de Montpellier, Etablissement Français du Sang, Inserm, Montpellier, France
[2]Department of Food Safety, Nutrition and Veterinary Public Health, Istituto Superiore di Sanita, Rome, Italy
[3]Inserm U 1127, CNRS UMR 7225, Sorbonne Universités, UPMC Université Paris 06 UMR S 1127, Institut du Cerveau et de la Moelle épinière, Paris, France
[4]Tanz Centre for Research in Neurodegenerative Diseases and Department of Biochemistry, University of Toronto, Toronto, Canada
[5]IRMB, INM, Univ Montpellier, INSERM, CHU Montpellier, (LBPC-PPC), Montpellier, France

*Address correspondence to Daisy Bougard, daisy.bougard@efs.sante.fr.

Supplementary Table S1: Summary of patients included in the PMCA brain study

Id	Classification	Gender	Age	Diagnosis	Disease duration
#1	MM1	F	78	Definite sCJD	3 months
#2	MM1	F	65	Definite sCJD	3 months
#3	MM1	F	52	Definite sCJD	2 months
#4	MM2	M	67	Definite sCJD	17 months
#5	MM2	M	68	Definite sCJD	13 months
#6	MV1	M	62	Definite sCJD	17 months
#7	MV1	M	74	Definite sCJD	2 months
#8	MV2	F	58	Definite sCJD	10 months
#9	MV2	F	64	Definite sCJD	16 months
#10	VV1	M	63	Definite sCJD	5 months
#11	VV1	F	33	Definite sCJD	19 months
#12	VV2	F	76	Definite sCJD	7 months
#13	VV2	M	60	Definite sCJD	6 months
#14	VV2	M	75	Definite sCJD	4 months
#15	MM2B	na	na	Definite vCJD	na

Supplementary Table S2: Summary of patients included in the CSF study

Id	Classification	Gender	Age	Diagnosis	CSF 14-3-3
§1	MM	M	86	Probable sCJD	Positive
§2	MM	F	75	Probable sCJD	Positive
§3	MM1	M	67	Definite sCJD	Positive
§4	MM1	M	68	Definite sCJD	Positive
§5	MM2	M	65	Definite sCJD	Positive
§6	VV2	M	77	Definite sCJD	Positive
§7	VV	F	70	Probable sCJD	Positive
§8	VV	M	79	Probable sCJD	Positive
§9	MM2B	F	27	Definite vCJD	Negative

Supplementary Figure S1: PMCA amplification of sCJD MM1 in TgMet, BV and TgBV

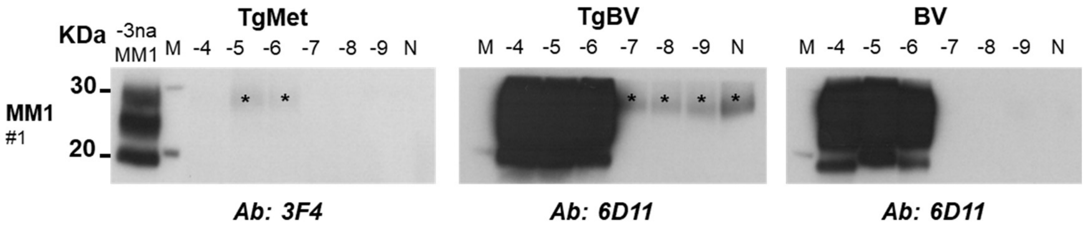

Serial dilutions from 10^{-4} to 10^{-9} of the MM1 subtype sCJD homogenate (#1) were amplified using TgMet, TgBV or BV substrates. After 4 rounds of PMCA, the PrP^{TSE} signal was analyzed by western blot after proteinase K digestion using 3F4 or 6D11 antibodies as indicated. For each sample, 20 µL of the product were loaded onto the gel. -3na MM1 refers to non-amplified material (no PMCA) obtained from the 10^{-3} dilution (w/v) of the initial infectious brain sample of the MM1 subtype sCJD patient #1. N refers to unseeded substrate amplified in the same conditions. The asterisk indicates a faint signal from incomplete PrP^{C} digestion. M indicates the typical molecular mass of PrP^{res} in the range of 20 to 30 kDa.

Supplementary uncropped original blots

Figure 1

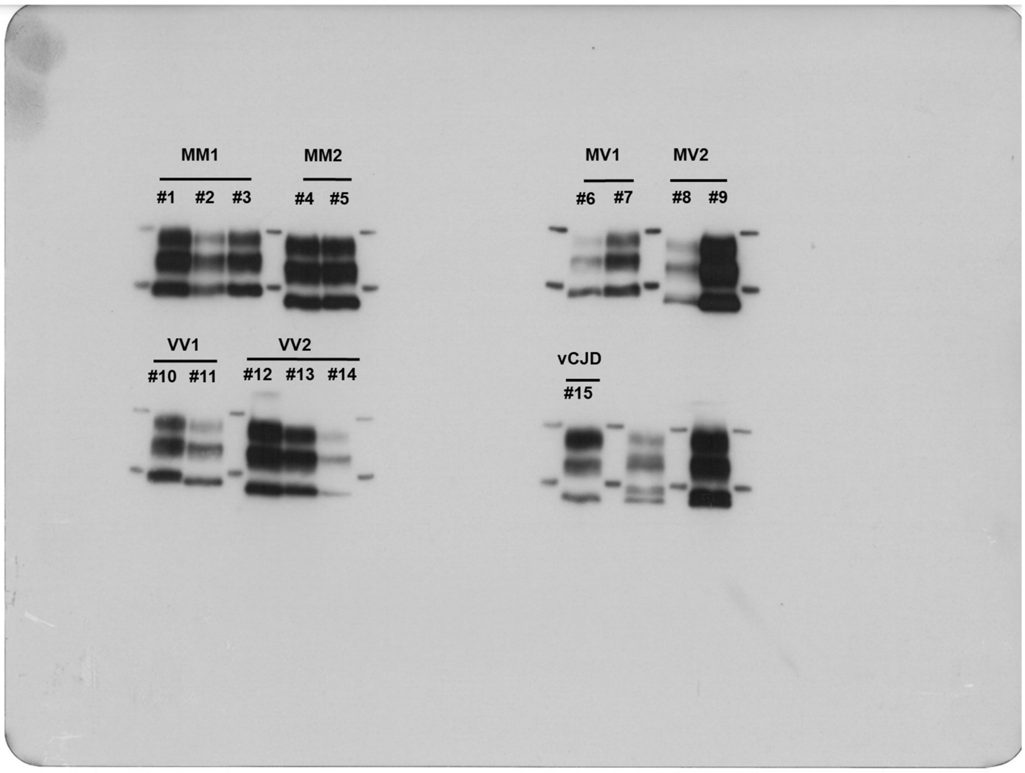

Figure 2

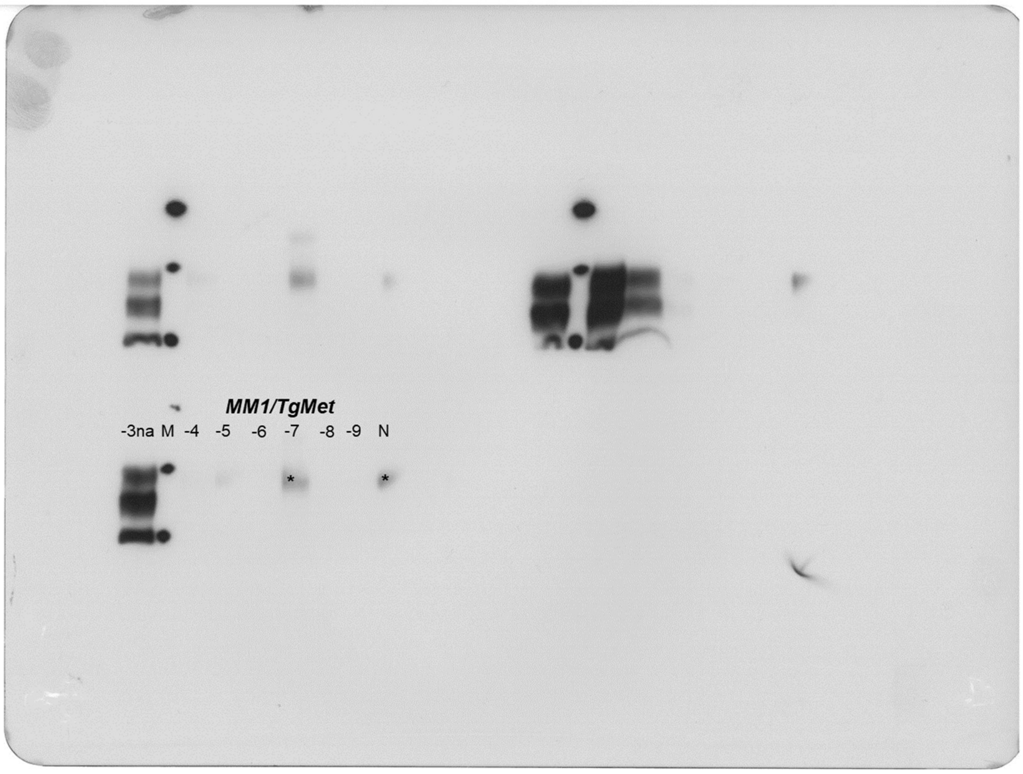

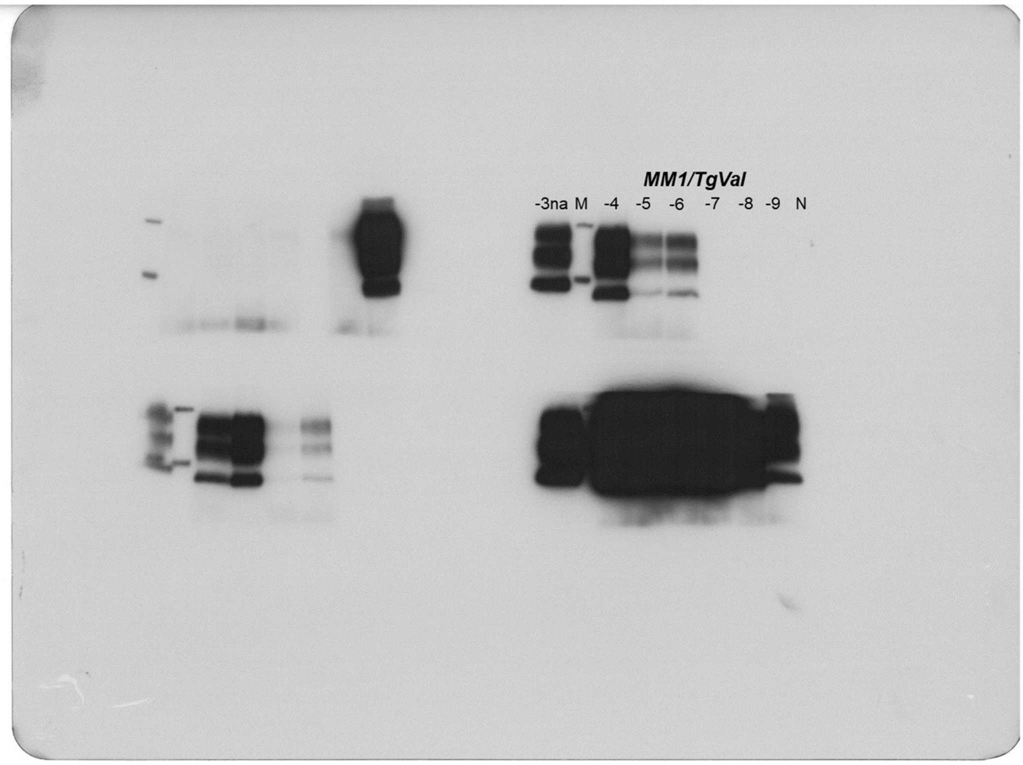
MM1/TgVal
-3na M -4 -5 -6 -7 -8 -9 N

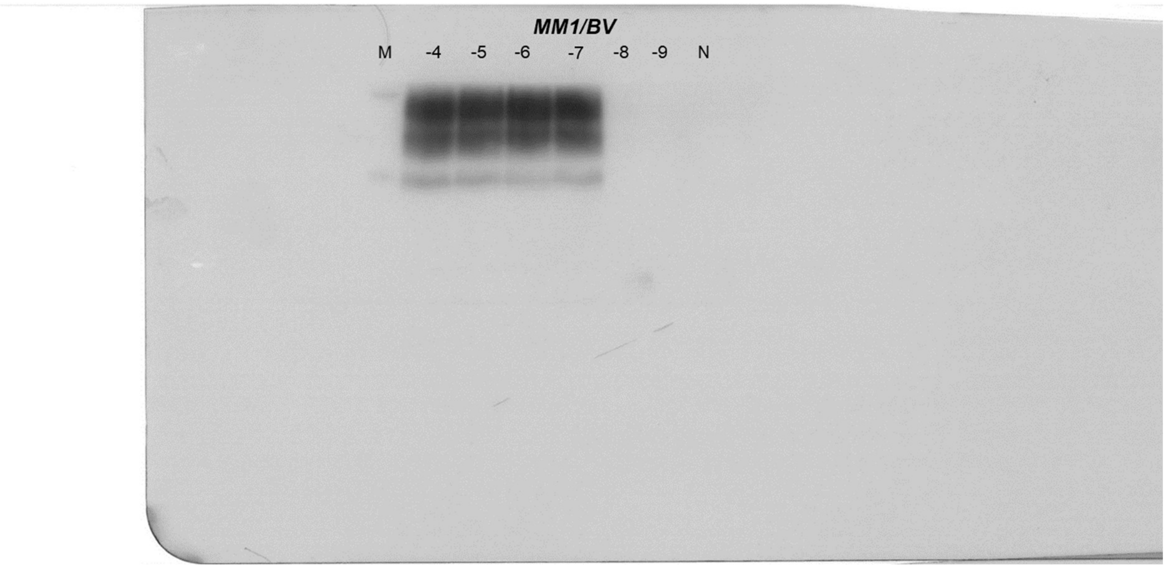
MM1/BV
M -4 -5 -6 -7 -8 -9 N

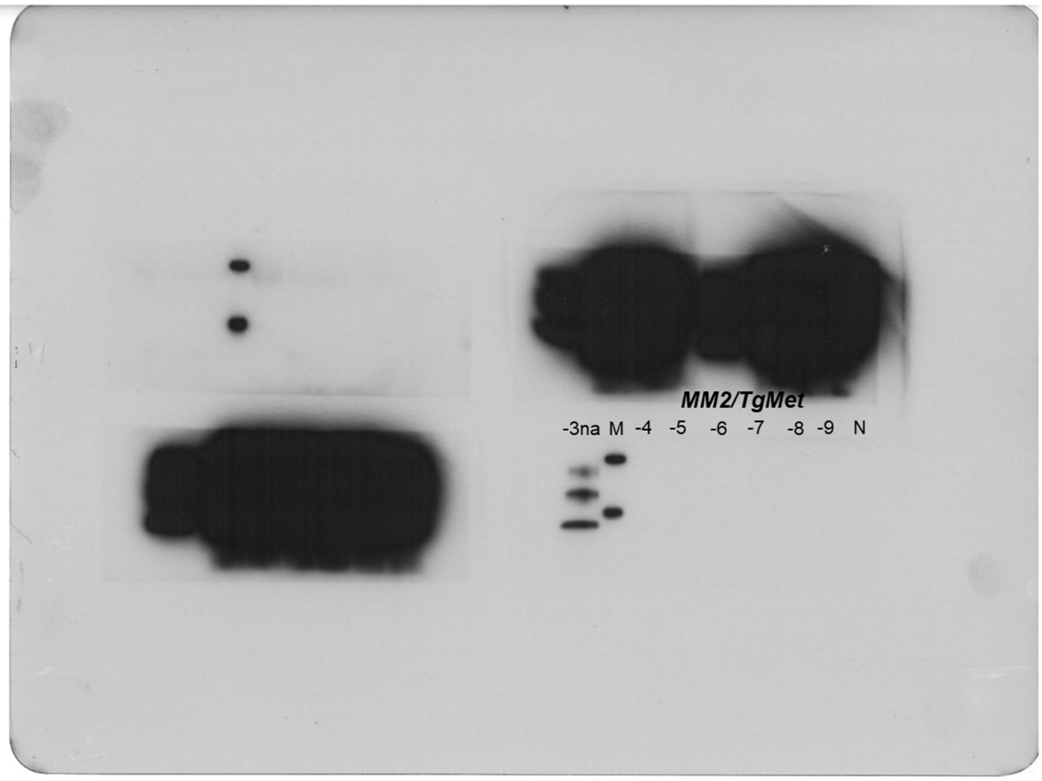
MM2/TgMet
-3na M -4 -5 -6 -7 -8 -9 N

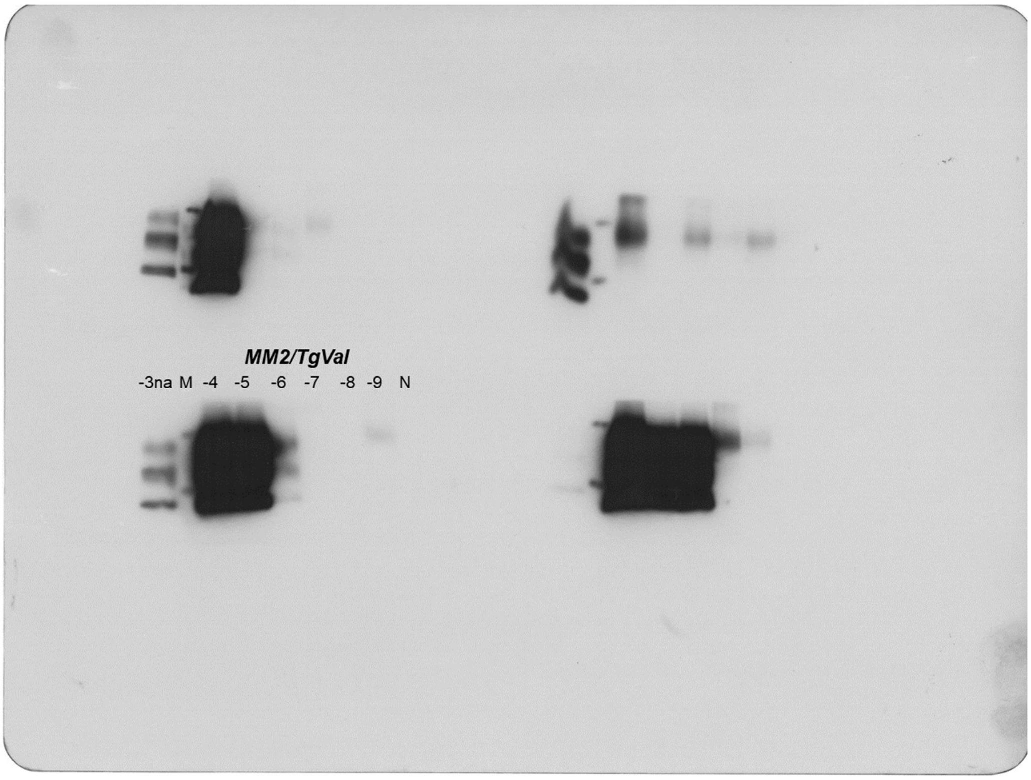
MM2/TgVal
-3na M -4 -5 -6 -7 -8 -9 N

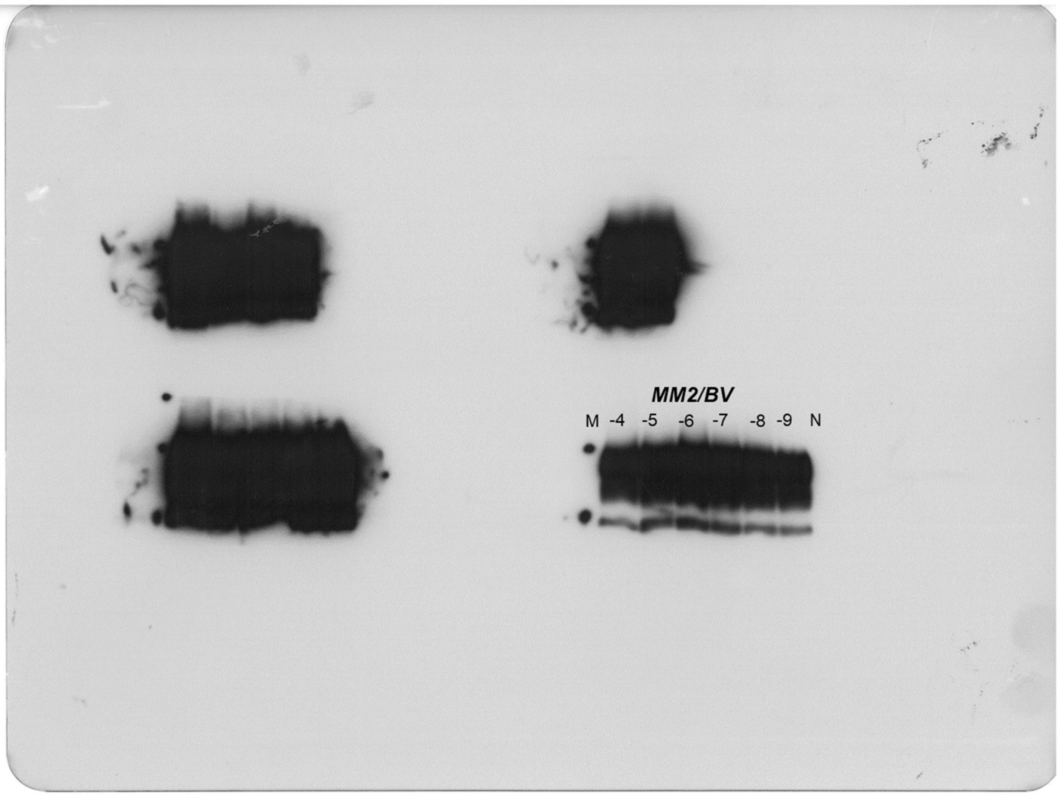
MM2/BV
M -4 -5 -6 -7 -8 -9 N

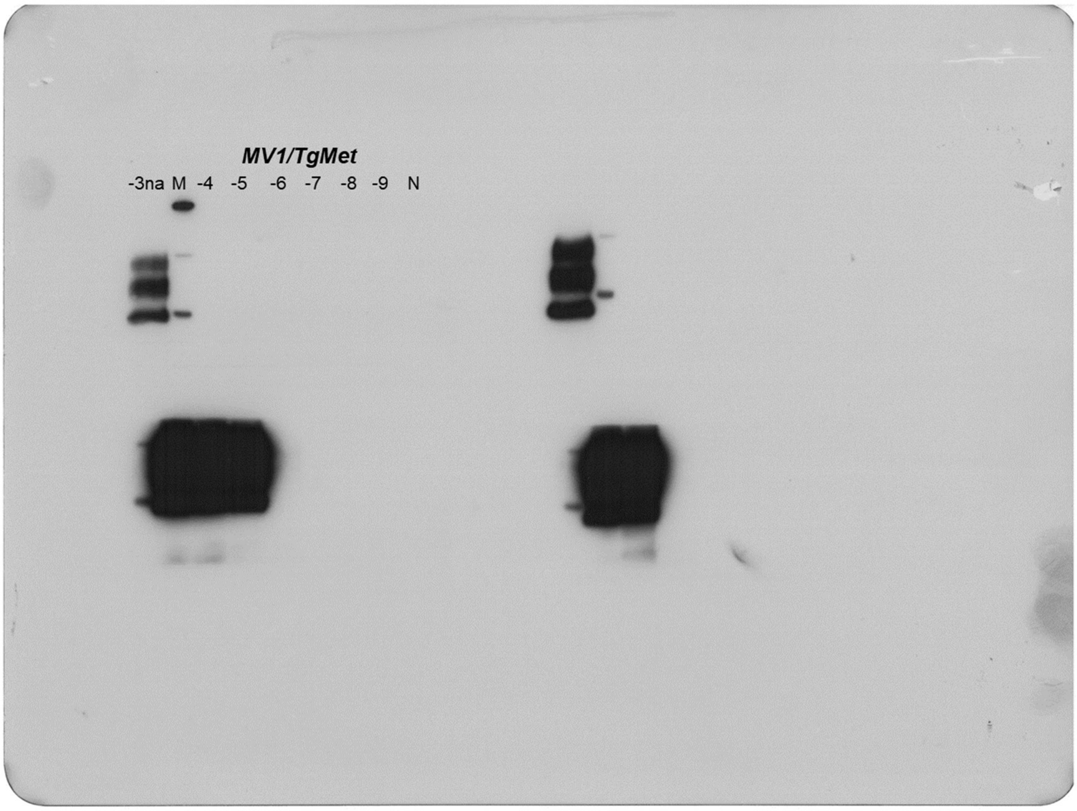
MV1/TgMet
-3na M -4 -5 -6 -7 -8 -9 N

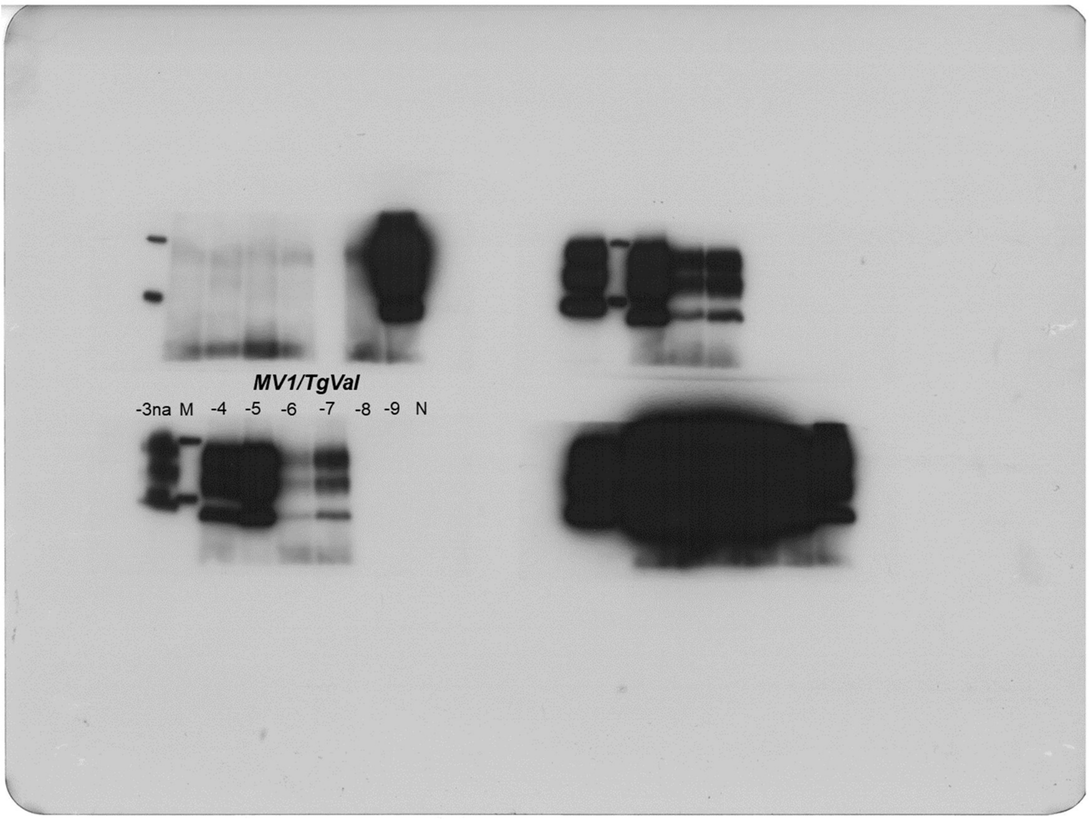
MV1/TgVal
-3na M -4 -5 -6 -7 -8 -9 N

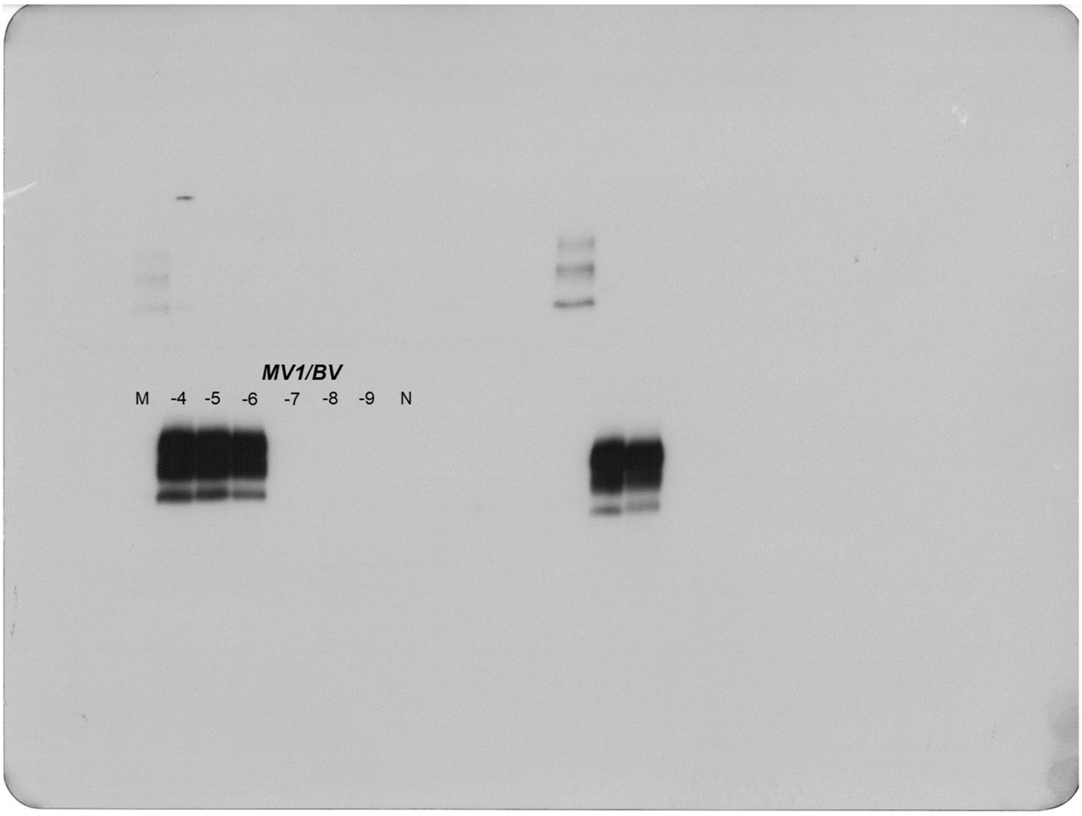
MV1/BV
M -4 -5 -6 -7 -8 -9 N

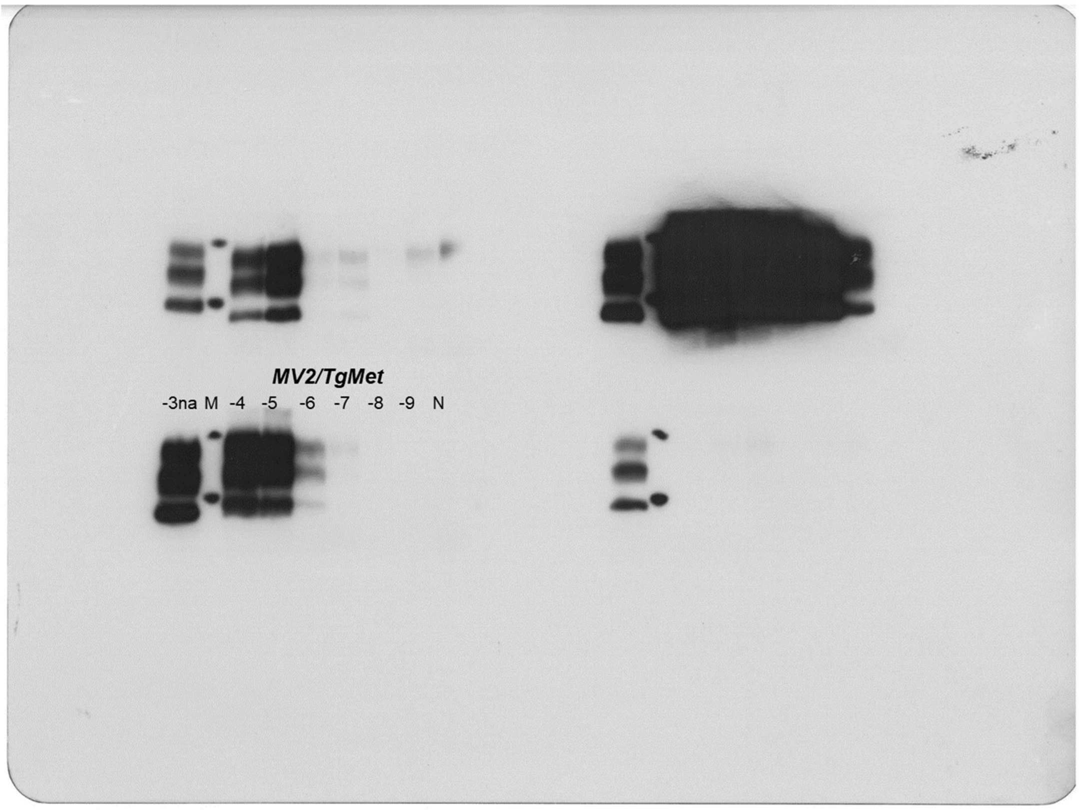
MV2/TgMet
-3na M -4 -5 -6 -7 -8 -9 N

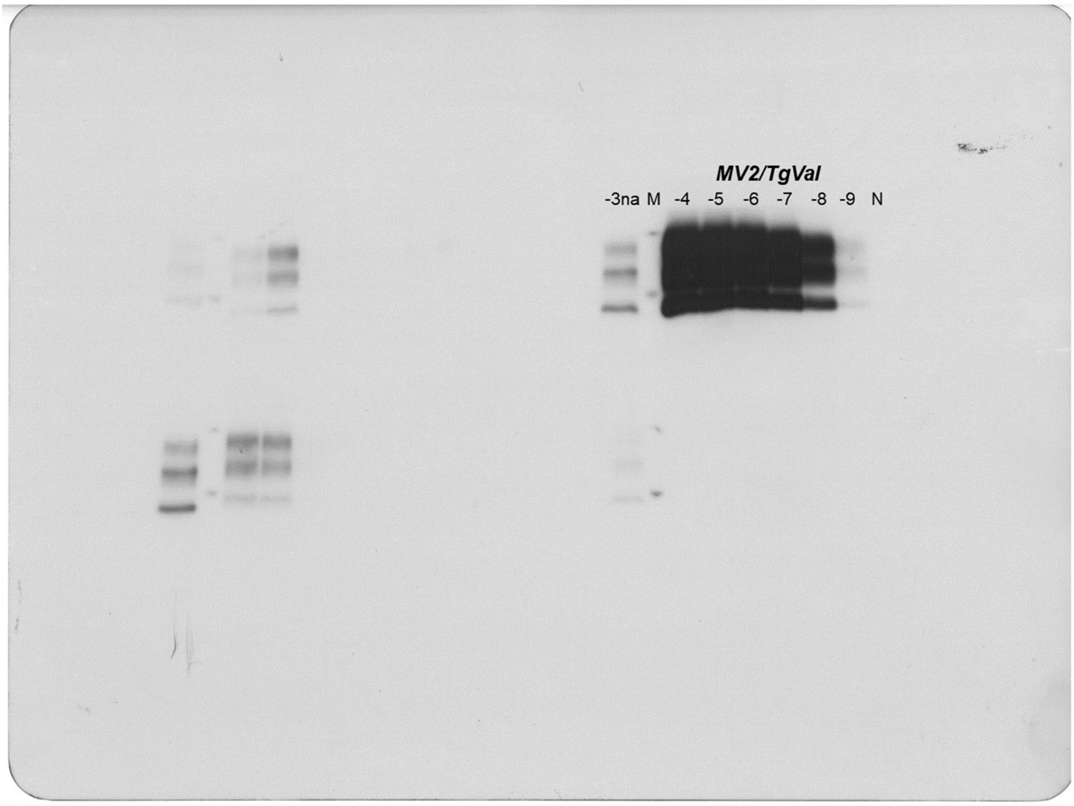
MV2/TgVal
-3na M -4 -5 -6 -7 -8 -9 N

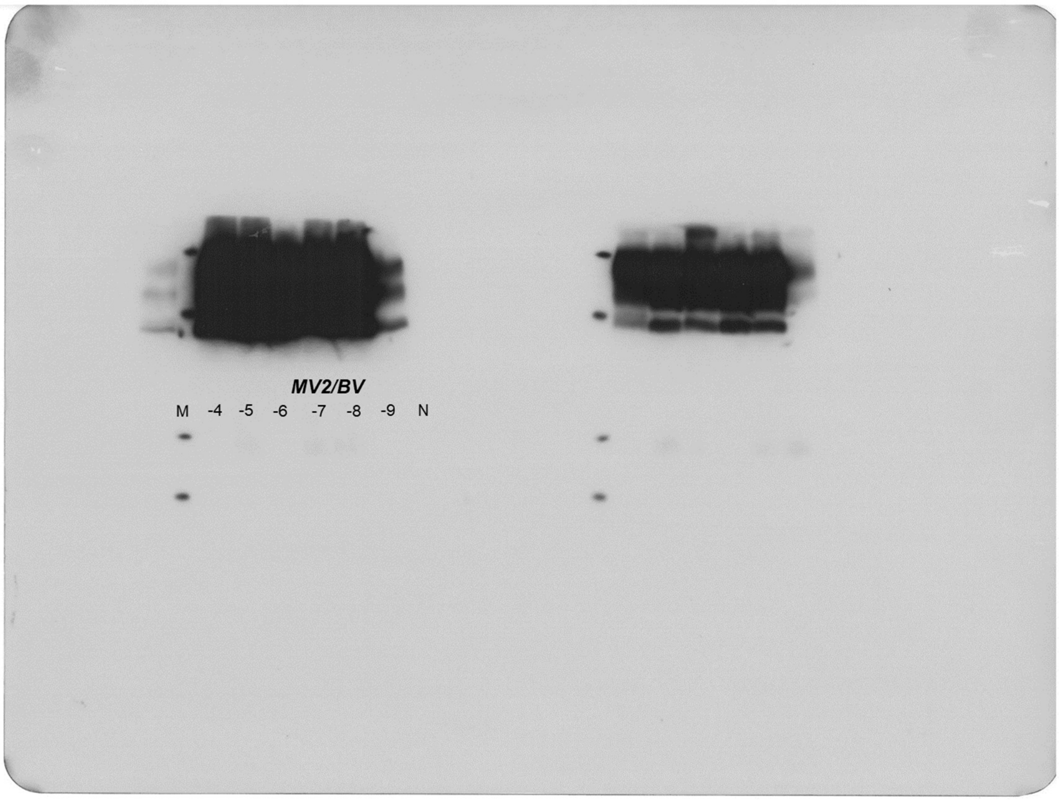
MV2/BV
M -4 -5 -6 -7 -8 -9 N

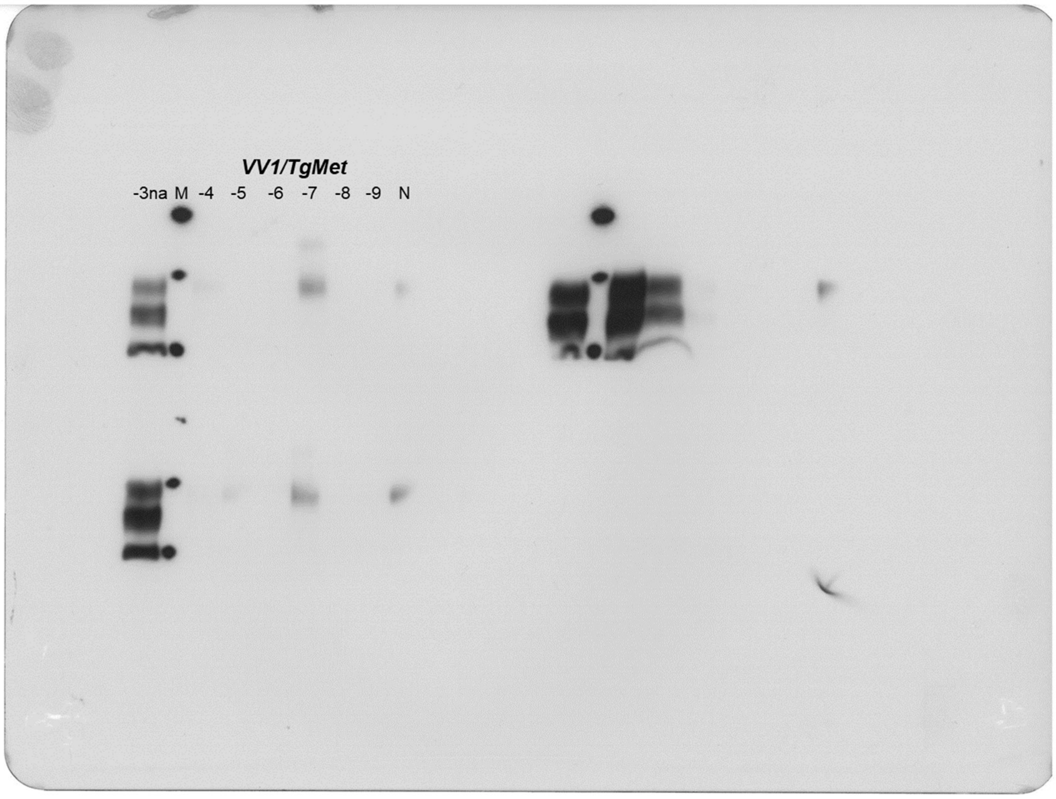
VV1/TgMet
-3na M -4 -5 -6 -7 -8 -9 N

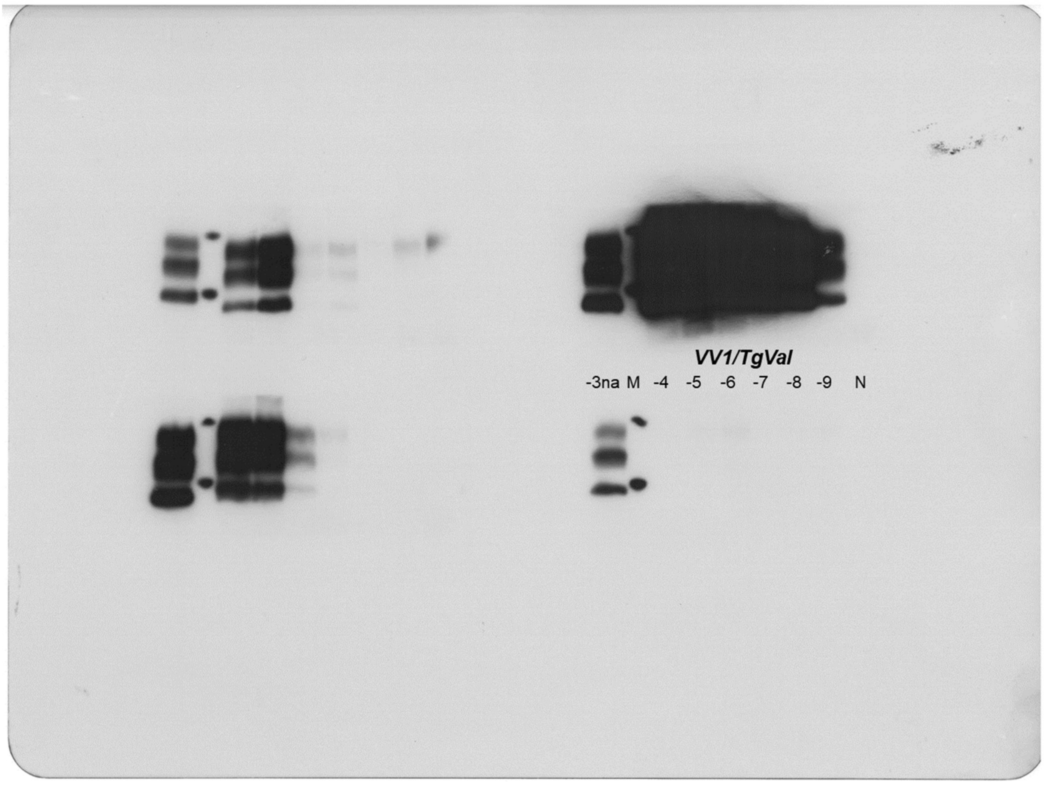
VV1/TgVal
-3na M -4 -5 -6 -7 -8 -9 N

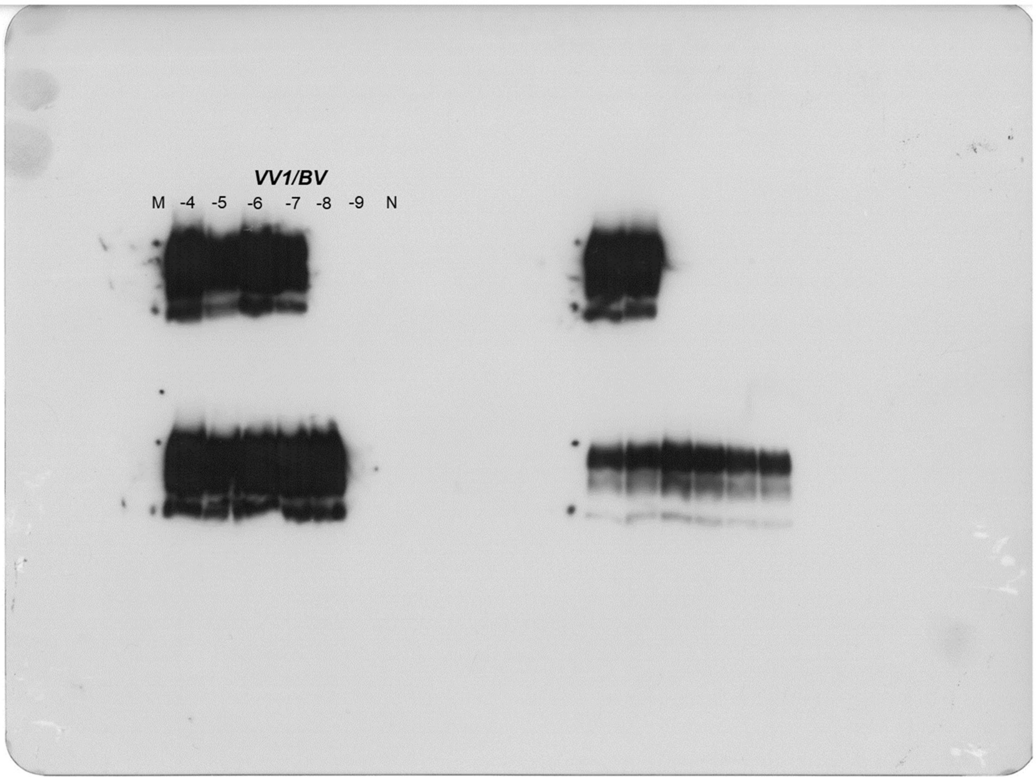
VV1/BV
M -4 -5 -6 -7 -8 -9 N

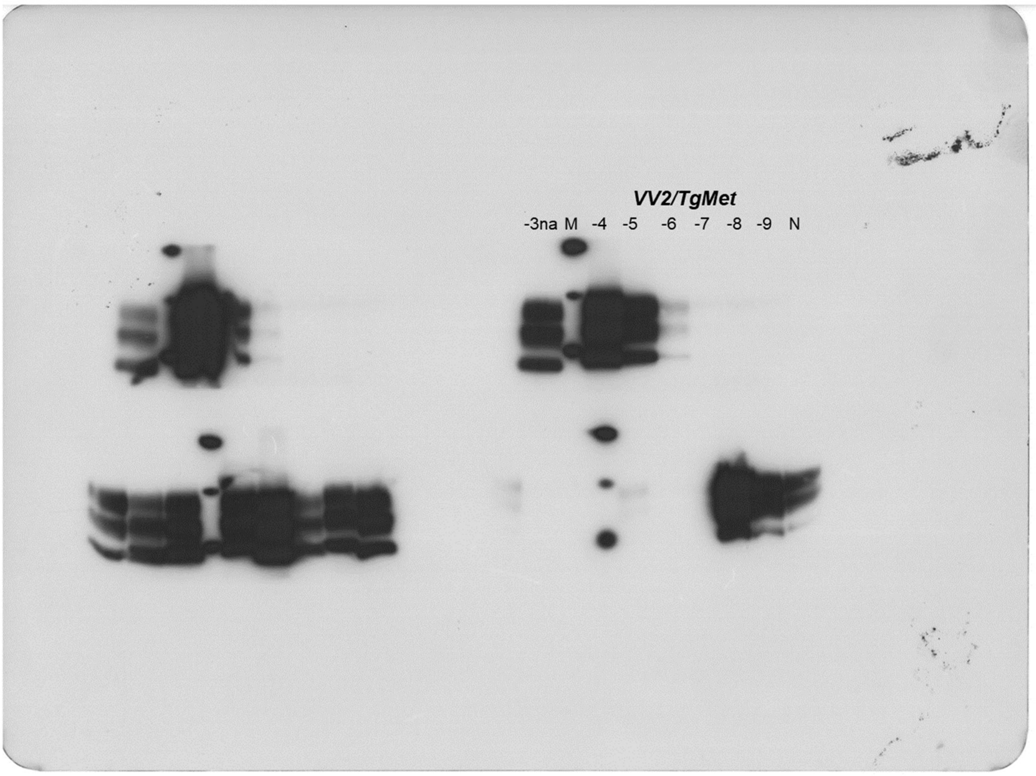
VV2/TgMet
-3na M -4 -5 -6 -7 -8 -9 N

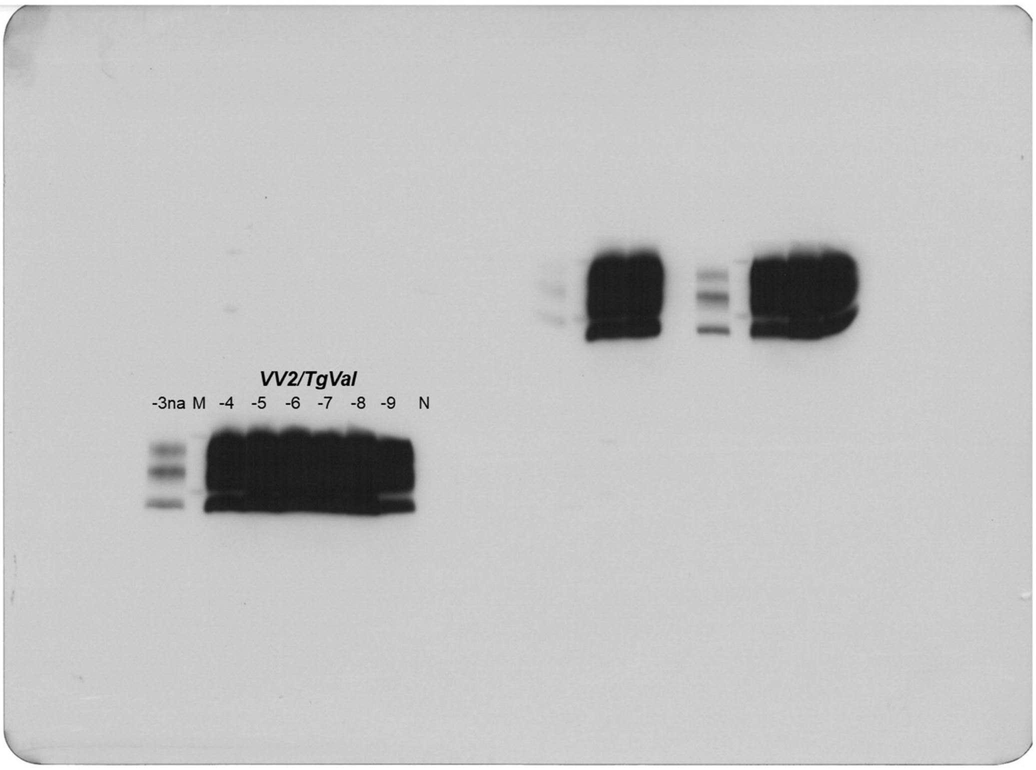
VV2/TgVal
-3na M -4 -5 -6 -7 -8 -9 N

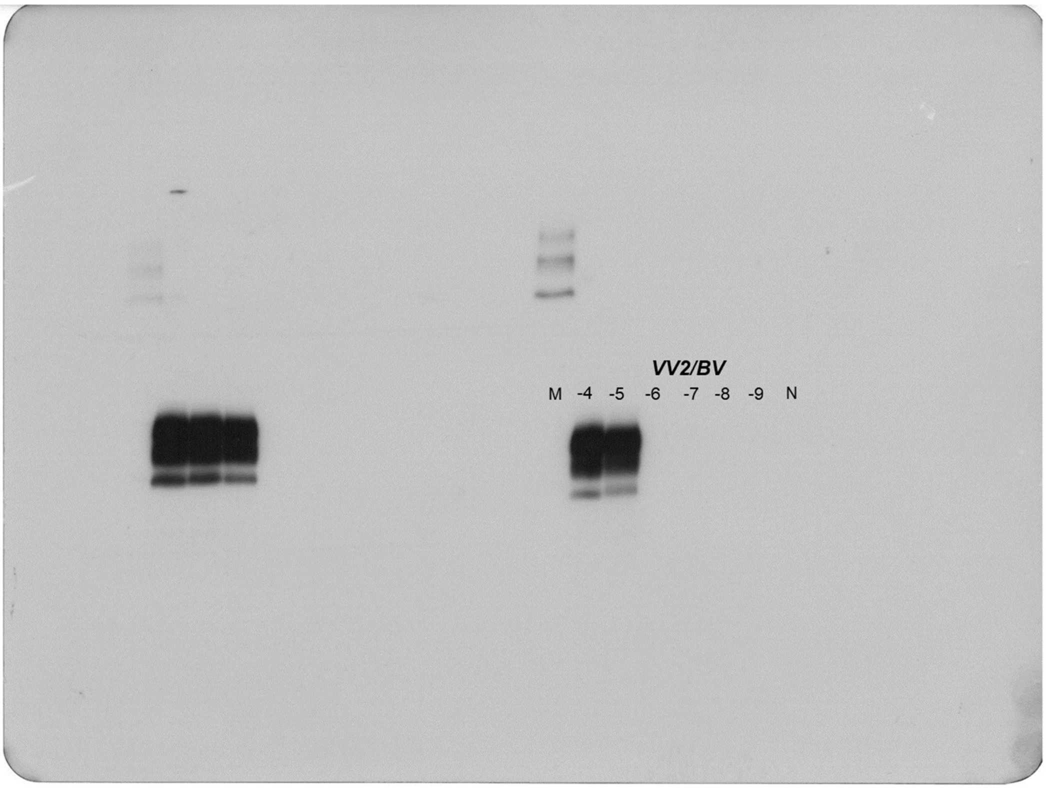

vCJD/TgMet

-3na M -4 -5 -6 -7 -8 -9 N

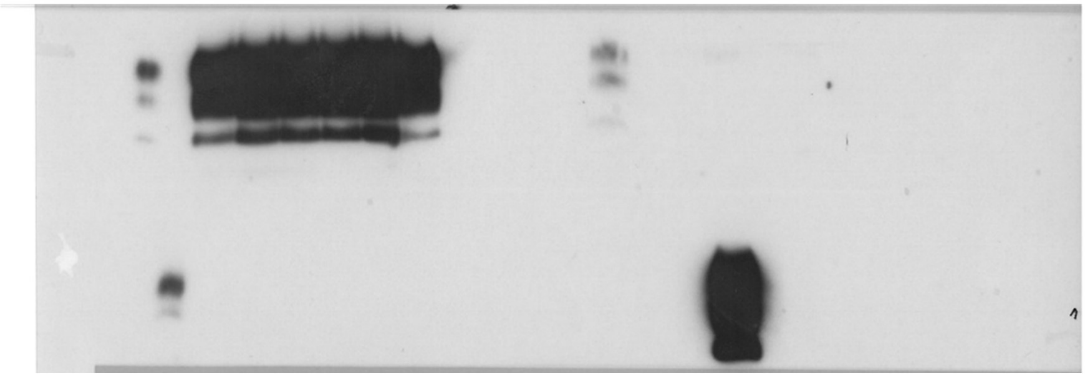

vCJD/TgVal

-3na M -4 -5 -6 -7 -8 -9 N

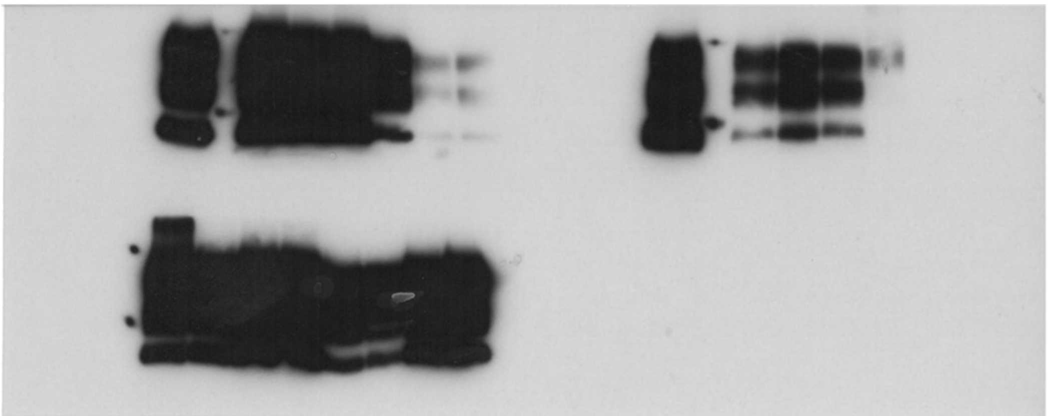

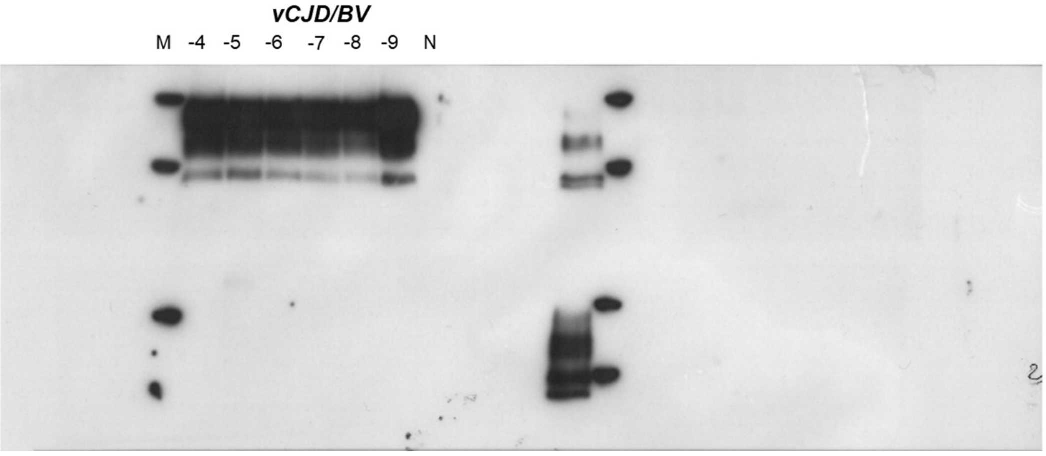
vCJD/BV
M -4 -5 -6 -7 -8 -9 N

Figure 3a

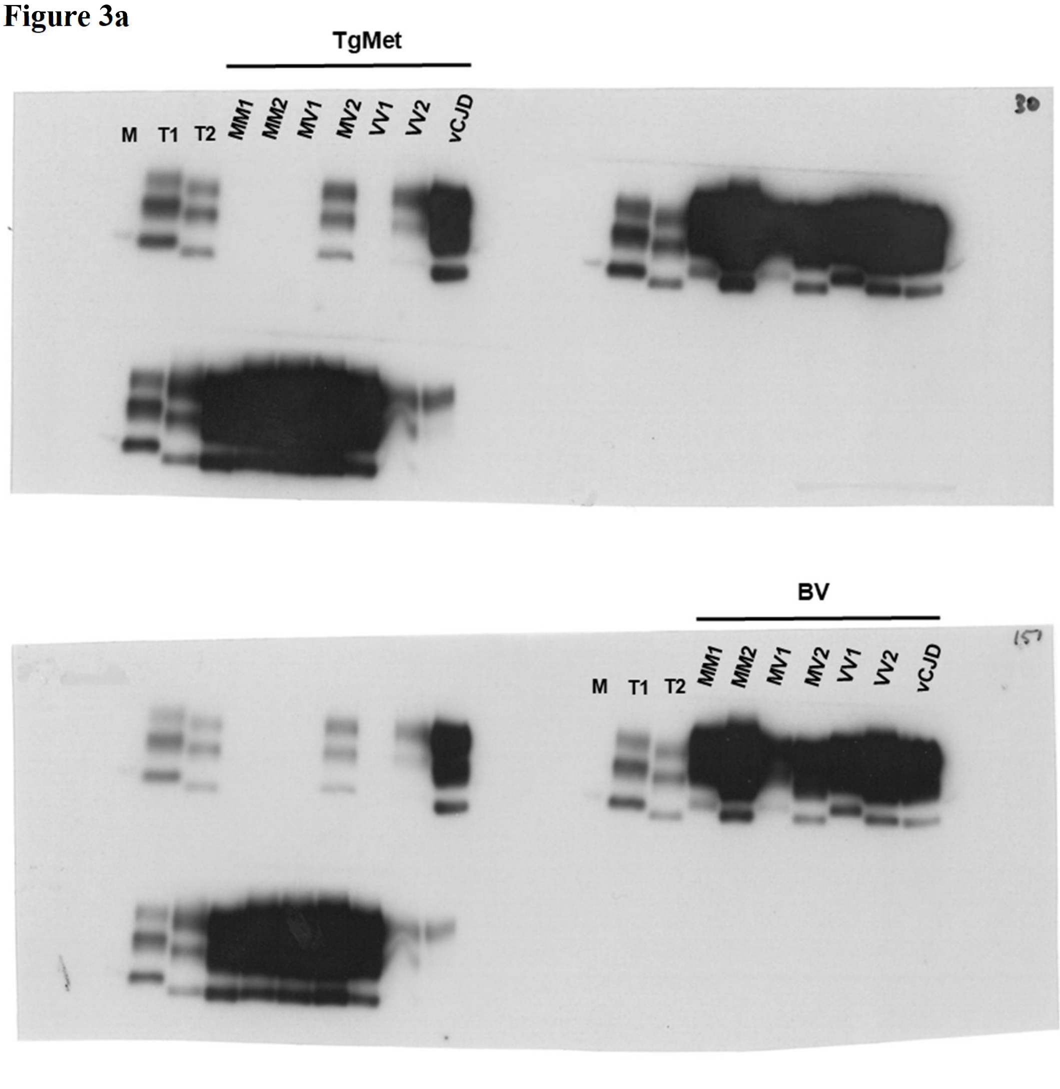
TgMet
M T1 T2 MM1 MM2 MV1 MV2 VV1 VV2 vCJD
BV
M T1 T2 MM1 MM2 MV1 MV2 VV1 VV2 vCJD

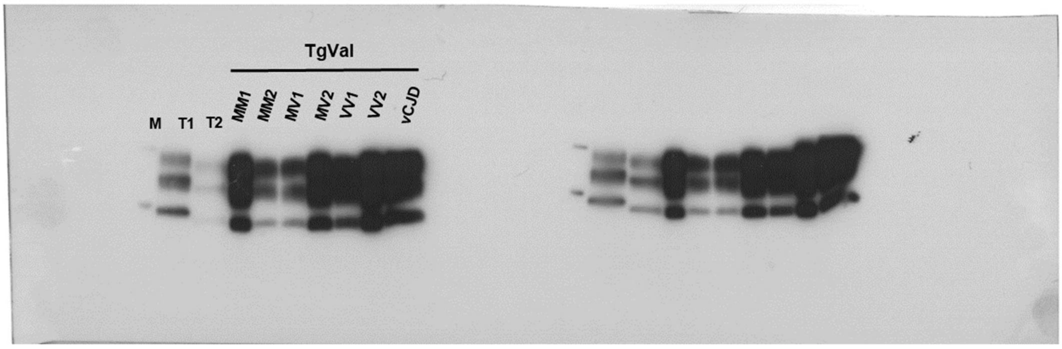
TgVal
M
T1
T2
MM1
MM2
MV1
MV2
VV1
VV2
vCJD

Figure 3b

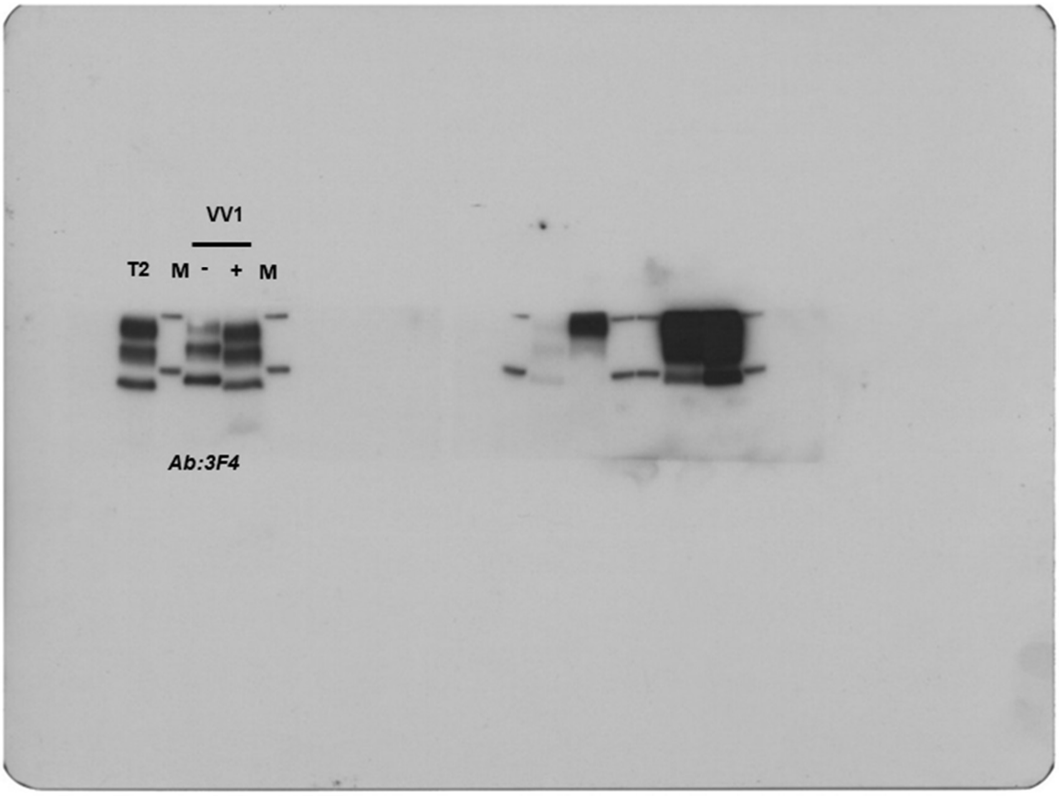
VV1
T2
M
-
+
M
Ab:3F4

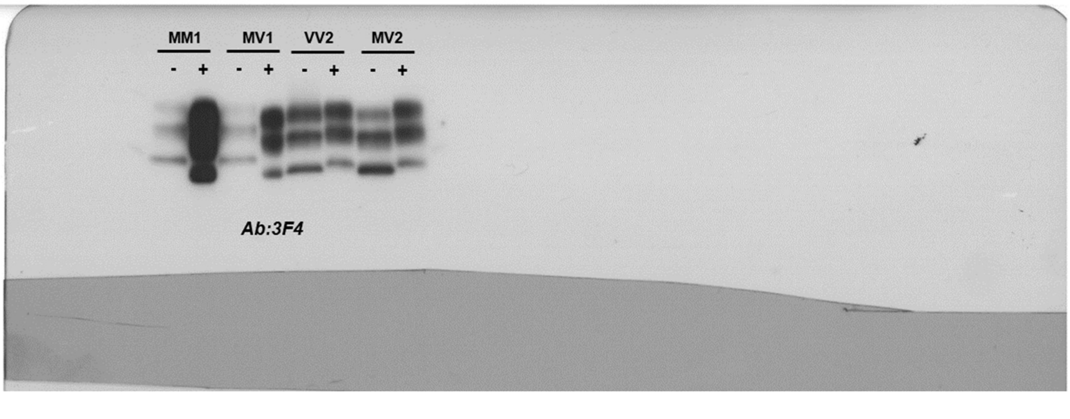
MM1
MV1
VV2
MV2
- +
- +
- +
- +
Ab:3F4

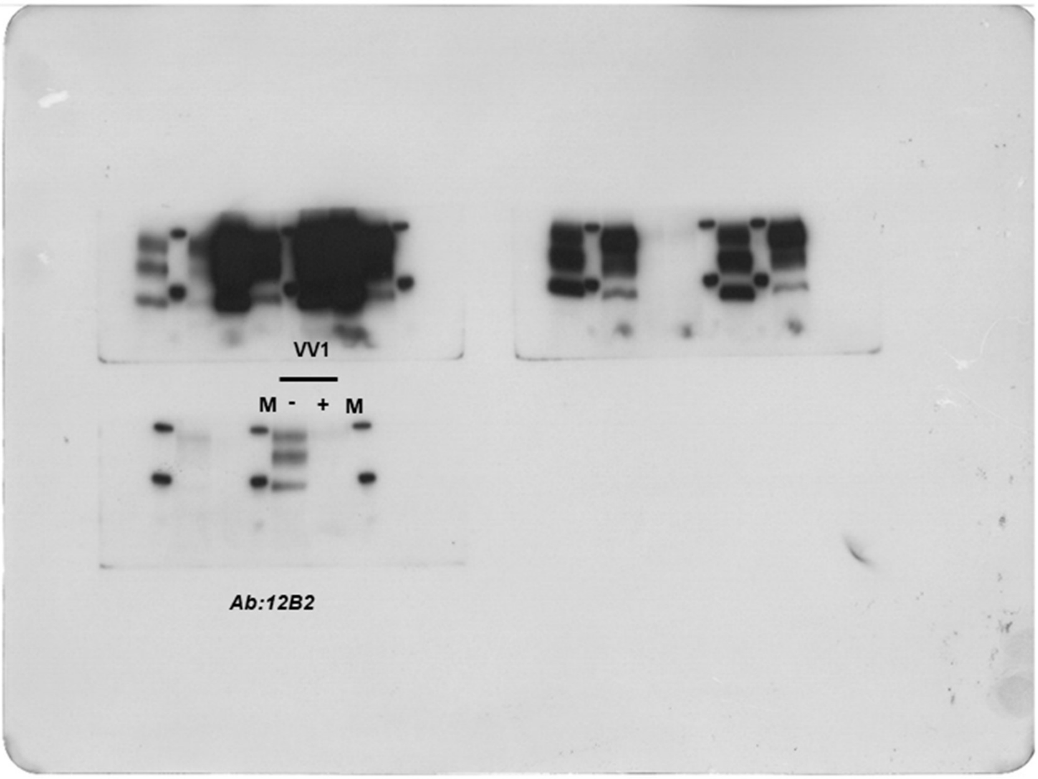
VV1
M - + M
Ab:12B2

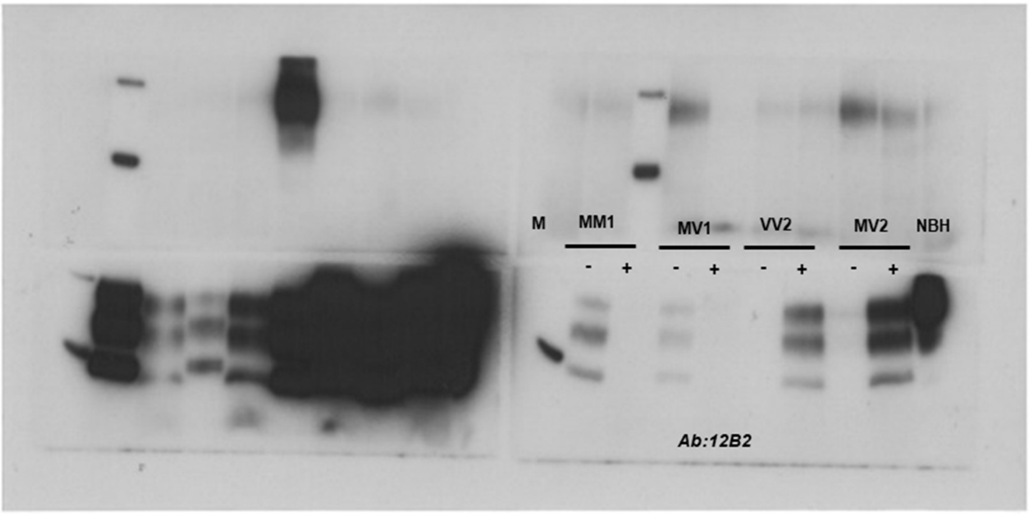
M
MM1
MV1
VV2
MV2
NBH
- +
- +
- +
- +
Ab:12B2

Figure 3c (digital imaging system)

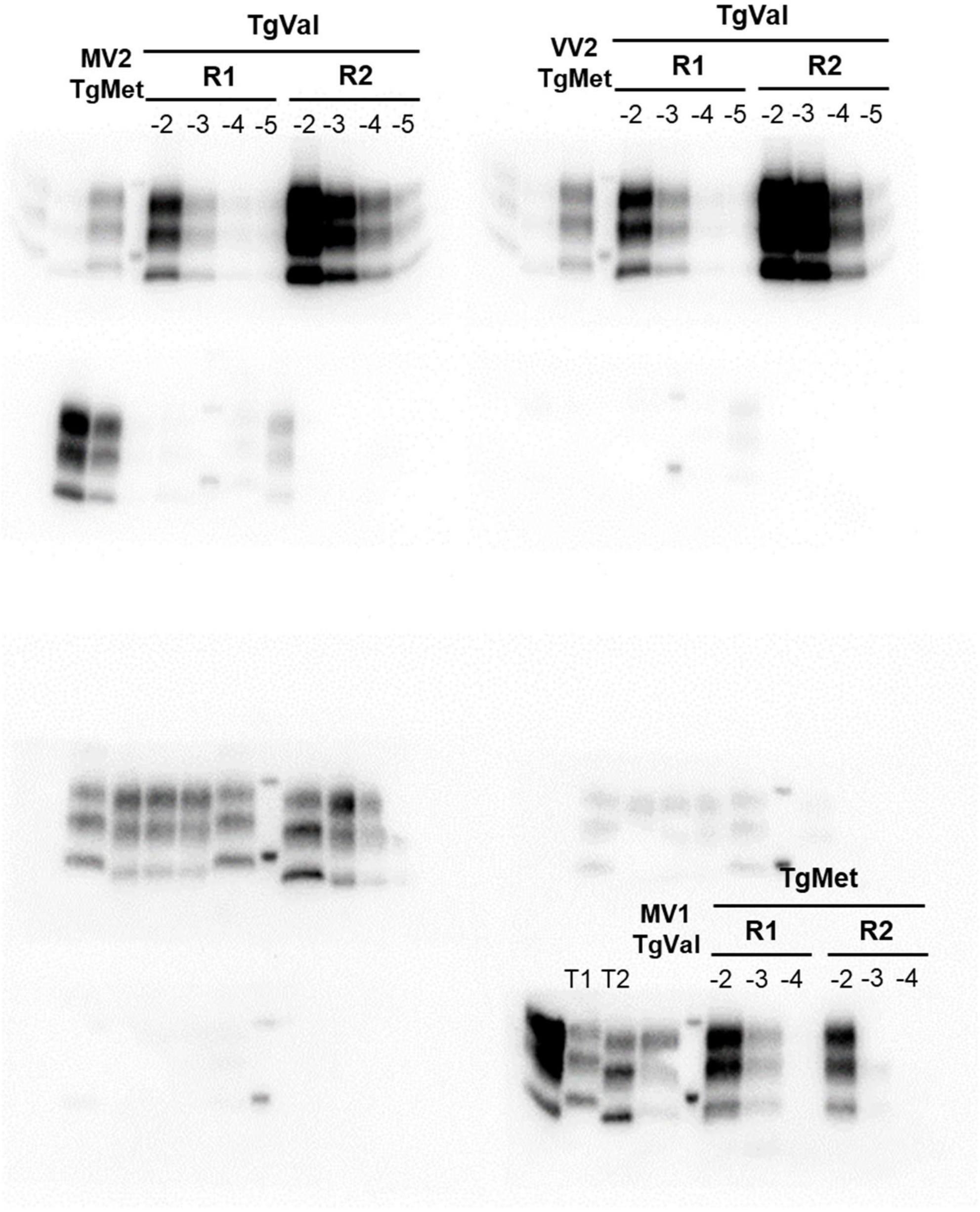

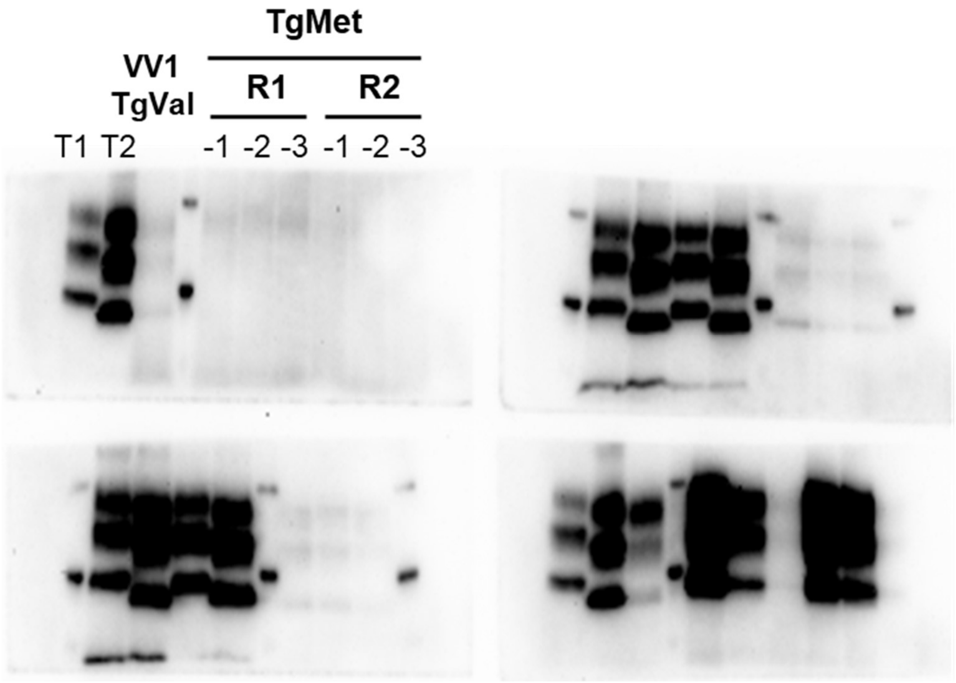
TgMet
VV1
TgVal
R1
R2
T1 T2
-1 -2 -3 -1 -2 -3

Figure 4

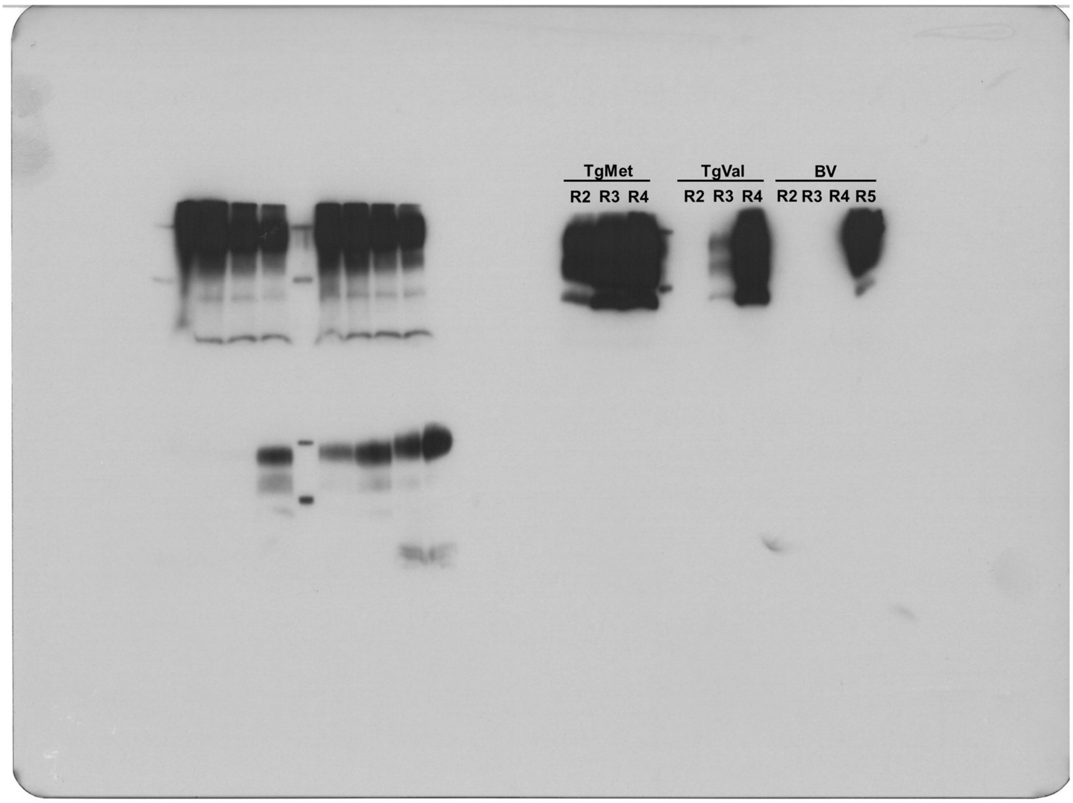
TgMet
TgVal
BV
R2 R3 R4
R2 R3 R4
R2 R3 R4 R5

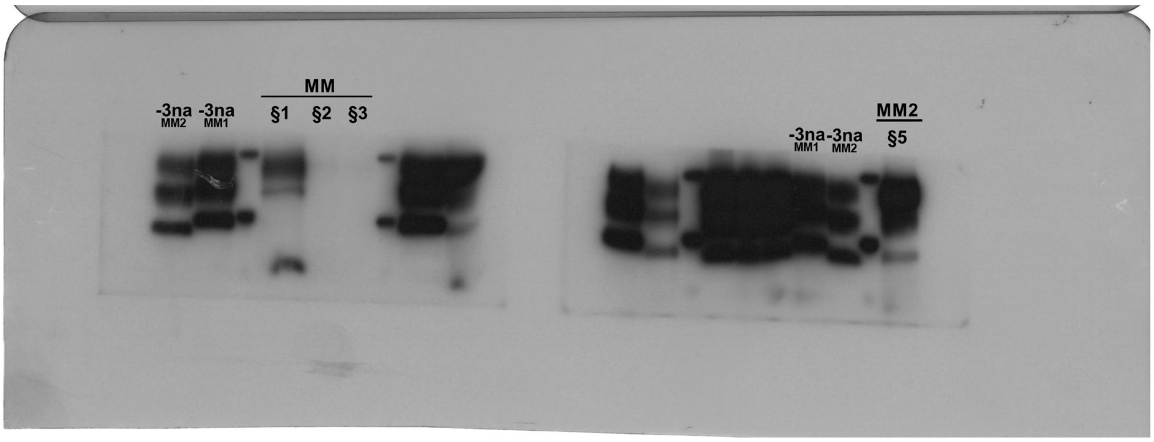
MM
-3na MM2
-3na MM1
§1
§2
§3
MM2
-3na MM1
-3na MM2
§5

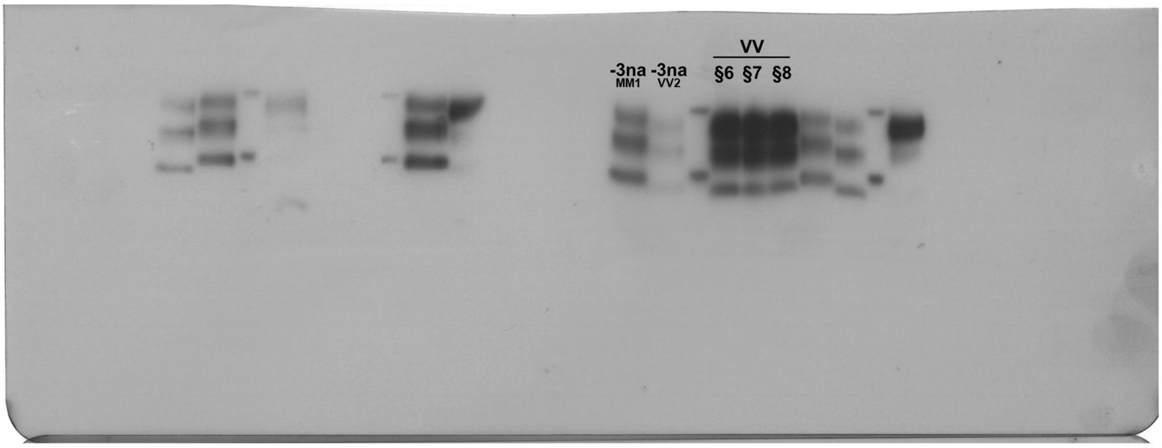
VV
-3na MM1
-3na VV2
§6
§7
§8

Figure S1

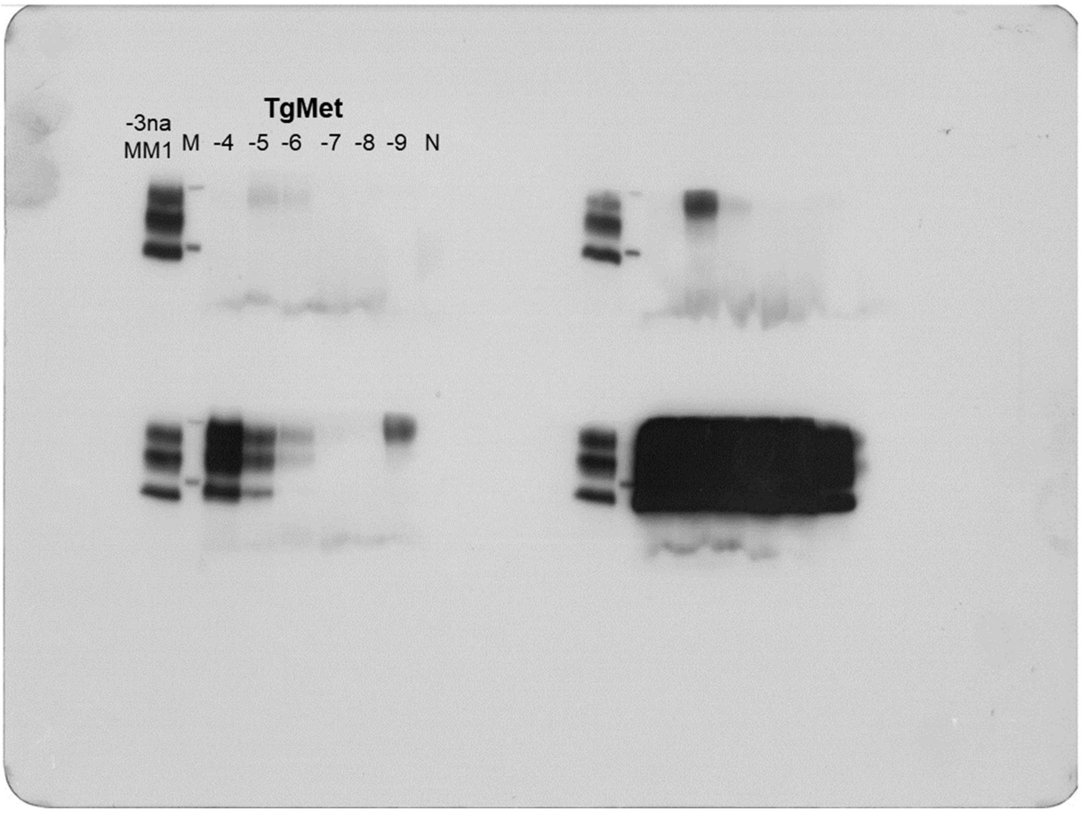

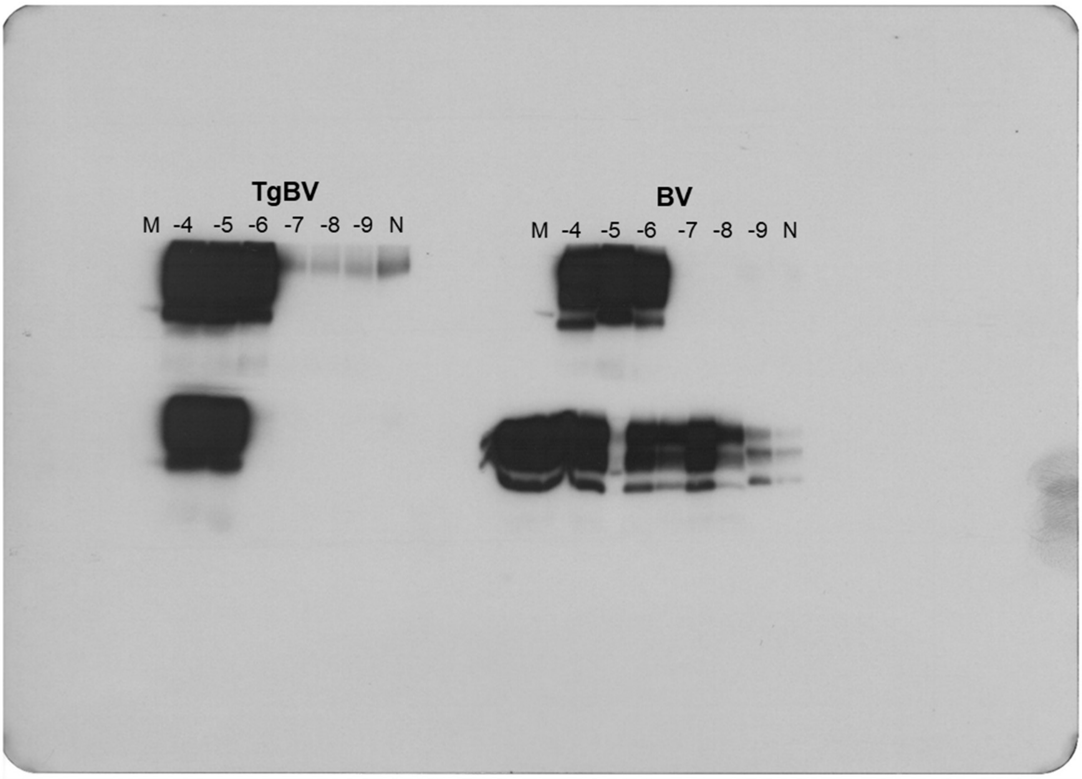

Supplement: Supplementary file 1 — Supplementary Information. [file 41598_2021_83630_MOESM1_ESM.pdf]
